# Aerobic, muscle-strengthening, and flexibility physical activity and risks of all-cause and cause-specific mortality: a population-based prospective cohort of Korean adults

**DOI:** 10.1186/s12889-023-15969-1

**Published:** 2023-06-14

**Authors:** Yoonkyoung Cho, Hajin Jang, Sohyeon Kwon, Hannah Oh

**Affiliations:** 1 Interdisciplinary Program in Precision Public Health, College of Health Science, Graduate School of Korea University, Seoul, Republic of Korea; 2 Department of Epidemiology, Johns Hopkins Bloomberg School of Public Health, Baltimore, MD USA; 3 Department of Health Policy and Management, College of Health Science, Korea University, Seoul, Republic of Korea; 4 Korea University, 145 Anam-ro, Seongbuk-gu Hana Science Bldg B358, Seoul, Republic of Korea

**Keywords:** Physical activity, Exercise, Death, Mortality, Lifestyle, Asian

## Abstract

**Background:**

Studies have shown that aerobic and muscle-strengthening physical activities reduce mortality risk. However, little is known about the joint associations of the two activity types and whether other type of physical activity, such as flexibility activity, can provide similar mortality risk reduction.

**Objectives:**

We examined the independent associations of aerobic, muscle-strengthening, and flexibility physical activities with all-cause and cause-specific mortality in a population-based prospective cohort of Korean men and women. We also examined the joint associations of aerobic and muscle-strengthening activities, the two physical activity types that are recommended by the current World Health Organization physical activity guidelines.

**Design:**

This analysis included 34,379 Korea National Health and Nutrition Examination Survey 2007–2013 participants (aged 20–79 years) with mortality data linkage through December 31, 2019. Engagement in walking, aerobic, muscle-strengthening, and flexibility physical activities was self-reported at baseline. Cox proportional hazards model was performed to estimate hazard ratios (HRs) and 95% confidence intervals (CIs), adjusting for potential confounders.

**Results:**

Flexibility physical activity (≥ 5 vs. 0 d/wk) was inversely associated with all-cause (HR [95% CI] = 0.80 [0.70–0.92]; *P*-trend < 0.001) and cardiovascular mortality (0.75 [0.55–1.03], *P*-trend = 0.02). Moderate- to vigorous-intensity aerobic physical activity (≥ 50.0 vs. 0 MET-h/wk) was also associated with lower all-cause (HR [95% CI] = 0.82 [0.70–0.95]; *P*-trend < 0.001) and cardiovascular mortality (0.55 [0.37–0.80]; *P*-trend < 0.001). Similar inverse associations were observed with total aerobic physical activity, including walking. Muscle-strengthening activity (≥ 5 vs. 0 d/wk) was inversely associated with all-cause mortality (HR [95% CI] = 0.83 [0.68–1.02]; *P*-trend = 0.01) but was not associated with cancer or cardiovascular mortality. Compared to participants meeting the highest guidelines for both moderate- to vigorous-intensity aerobic and muscle-strengthening physical activities, those not meeting in any guideline were associated with higher all-cause (1.34 [1.09–1.64]) and cardiovascular mortality (1.68 [1.00-2.82]).

**Conclusions:**

Our data suggest that aerobic, muscle-strengthening, and flexibility activities are associated with lower risk of mortality.

**Supplementary Information:**

The online version contains supplementary material available at 10.1186/s12889-023-15969-1.

## Introduction

Physical activity refers to any bodily movement produced by skeletal muscles that requires energy expenditure [1]. Physical activity includes various types of activity, such as aerobic, muscle-strengthening, and flexibility physical activities. Studies have shown that aerobic and muscle-strengthening physical activities are associated with lower risks of obesity [2, 3], hypertension [4–6], type 2 diabetes [7, 8], cancer [9, 10], and cardiovascular diseases [11, 12], the major causes of premature deaths in many countries [13, 14]. Based on the findings that support the health benefits of physical activity, the current World Health Organization (WHO) physical activity guideline recommends adults to engage in at least 150 min/wk of moderate-intensity aerobic physical activity (or ≥ 75 min/wk of vigorous-intensity aerobic physical activity) and more than 2 d/wk of muscle-strengthening physical activity [15].

Despite the large number of studies on physical activity, there are some remaining questions that have not been fully investigated. Because most studies focused on a single activity type, it is unclear whether engaging in recommended levels of both aerobic and muscle-strengthening activities provides additional benefits compared with meeting the guideline for one. Further, little is known about the association between physical activity and mortality beyond aerobic and muscle-strengthening activities and thus it is yet unclear whether other types of physical activity, such as flexibility physical activity, can also provide similar mortality risk reduction. While some populations (e.g., elderly, people with disabilities) can only perform flexibility physical activity, the current guideline does not provide a clear recommendation regarding flexibility physical activity. In some studies, flexibility physical activity, including stretching and yoga, was associated with reduced risk of injuries [16–18] and improved pulmonary function [19–21]. However, evidence is scarce for the association between flexibility physical activity and mortality.

In this study, we examined the associations of three different types of physical activity (aerobic, muscle-strengthening, and flexibility physical activities) with all-cause and cause-specific mortality in a population-based prospective cohort of Korean adults. We also investigated the joint associations of the two recommended physical activity types (aerobic and muscle-strengthening) according to the current physical activity guidelines.

## Methods

### Study population

This study was conducted among participants in the Korea National Health and Nutrition Examination Survey (KNHANES) 2007–2013 who agreed to mortality follow-up through December 31, 2019. The KNHANES is a nationally representative, cross-sectional survey conducted by the Korea Disease Control and Prevention Agency (KDCA) and the Korean Ministry of Health and Welfare in 1998, 2001, 2005, and annually since 2007 [22]. The survey consists of health examination, health interview, and nutrition survey. The survey collected information on demographic characteristics, health behaviors, anthropometric measures, and health history. All surveys collected comparable data using similar methods. All participants provided informed consent and the survey was approved by the Institutional Review Board of the KDCA and Korea University.

Among 38,944 KNHANES 2007–2013 participants aged 20–79 years, 38,943 participants agreed to mortality data linkage. Among 38,943 who agreed to mortality linkage, we excluded participants who were currently pregnant (n = 227), had a prior diagnosis of cancer or cardiovascular disease at baseline (n = 2,800), had missing information on any type of physical activity (n = 1,217), had an unreasonable range of aerobic physical activity (> 16 h/d when combined walking, moderate-intensity, and vigorous-intensity activities; n = 53), and provided invalid responses to the physical activity questionnaire (mismatching information between frequency and duration; n = 13). To reduce bias due to subclinical disease at baseline, we further excluded participants who died during the first year of follow-up (n = 94) and those who reported to have lied down all day due to health problems (n = 160). In total, 34,379 participants (14,704 men and 19,675 women) were included in the analysis (participant flowchart in Supplementary Fig. 1).

### Mortality assessment

Among 34,379 participants, a total of 1,622 deaths were identified by matching death certificates based on the resident registration number. The underlying cause of death was obtained using codes from the International Classification of Diseases, 10th version (ICD-10). We identified deaths from all causes, as well as cancers (C00-D48) (574 deaths) and cardiovascular disease (I00-I99) (338 deaths).

### Physical activity assessment

During health interview, participants were asked to report the frequency (d/wk) and duration (h and min/d) of moderate-intensity and vigorous-intensity aerobic physical activity (including occupational and recreational activities) they performed during the past week, using a questionnaire derived from the International Physical Activity Questionnaire (IPAQ) [23, 24]. Participants were also asked to report the frequency and duration of walking (of any speed and purpose). For aerobic physical activity, we calculated total metabolic equivalent (MET)-h/wk by multiplying the summed h/wk of each activity with its corresponding average MET values (walking = 3.3 METs, moderate-intensity = 4 METs, vigorous-intensity = 8 METs) [25, 26]. We then categorized total aerobic physical activity (combined walking, moderate-intensity and vigorous-intensity activities) into three groups (low, moderate, high) according to the IPAQ guideline [27]. The “high” category of total aerobic activity included participants with ≥ 3 days of vigorous-intensity activity achieving total aerobic activity of ≥ 25 MET-h/wk; or ≥ 7 days of any combination of walking, moderate-intensity, or vigorous-intensity activities achieving total aerobic physical activity of ≥ 50 MET-h/wk. The “moderate” category included participants who satisfied at least one of the following three criteria: (1) ≥ 3 d/wk of vigorous-intensity activity of at least 20 min/d; (2) ≥ 5 d/wk of moderate-intensity activity and/or walking of at least 30 min/d; or (3) ≥ 5/wk of any combination of walking, moderate-intensity, or vigorous-intensity activities achieving total aerobic physical activity of ≥ 10 MET-h/wk. The “low” category included participant who were not meeting criteria for “moderate” and “high” categories. Additionally, because specific information on walking speed was not collected, we also separately estimated MET-h/wk of moderate- to vigorous-intensity aerobic physical activity after excluding walking. The WHO physical activity guideline [15] recommends engaging in at least 10 MET-h/wk of moderate- to vigorous-intensity aerobic activity for disease prevention and at least 20 MET-h/wk for additional health benefits. Based on the WHO guideline, we categorized moderate- to vigorous-intensity aerobic activity into six groups (0, 0.1–4.9, 5.0-9.9, 10.0-19.9, 20.0-49.9, ≥ 50.0 MET-h/wk). We included additional categories beyond those suggested by the WHO cutpoints to examine the dose-response relationship. For flexibility (e.g., stretching, free gymnastics) and muscle-strengthening (e.g., push-up, sit-up, weight-lifting using dumbbells or iron bars) physical activities, participants were asked to report the number of days (0, 1, 2, 3, 4, ≥ 5) they engaged in the activity during the past week. After collapsing categories with small samples, participants were categorized into five groups (0, 1, 2, 3–4, ≥ 5 d/wk). To examine the joint association of aerobic and muscle-strengthening activities, we categorized participants into groups according to their satisfaction with the physical activity guidelines: not meeting any guideline, meeting at least one guideline (aerobic or muscle-strengthening), and meeting both guidelines.

### Prevalent health conditions

During the baseline health interview, participants reported a prior diagnosis of type 2 diabetes, hypertension, and dyslipidemia. At baseline health examination, glycated hemoglobin (HbA1c), fasting blood glucose, blood triglyceride, blood cholesterol, and blood pressure were measured by medical staff, following standardized protocols. We defined prevalent type 2 diabetes as those who reported a prior diagnosis of type 2 diabetes, reported current use of diabetic medications, or had fasting blood glucose concentration of 126 mg/dL or higher or HbA1c of 6.5% or higher at baseline. Prevalent hypertension was defined as participants who reported a prior diagnosis of hypertension, reported current use of antihypertensive medications, or had diastolic blood pressure of 90 mmHg or higher or systolic blood pressure of 40 mmHg or higher at baseline. Prevalent dyslipidemia was defined as participants who satisfied at least one of the following criteria: (1) reported a prior diagnosis of dyslipidemia; (2) reported current use of medication or treatment; (3) had total cholesterol concentration of 240 mg/dL or higher; (4) had LDL-cholesterol concentration of 160 mg/dL or higher; (5) had HDL-cholesterol concentration of 40 mg/dL or lower; or (5) had triglyceride concentration of 200 mg/dL or higher. For each participant, we also summed the number (0–3) of prevalent health conditions: type 2 diabetes, hypertension, and dyslipidemia.

### Statistical analysis

From the baseline survey in 2007–2013, participants were followed up to the date of death or the end of follow-up on December 31, 2019. By using age as a time scale and the year of baseline examination as strata, Cox proportional hazards models were used to estimate hazard ratios (HRs) and 95% confidence intervals (CIs) for the associations between physical activity (aerobic, muscle-strengthening, flexibility) and mortality (all-cause as well as cancer- and cardiovascular disease-specific). All analyses accounted for the complex sampling design using a robust variance estimator. Multivariable models included potential confounders: sex, residence, education, occupation, household income, marital status, smoking status, alcohol drinking, and self-rated health (Model 1). In Model 2, we additionally adjusted for measured baseline body mass index (BMI), a potential mediator. In Model 3, we additionally adjusted for prevalence of type 2 diabetes, hypertension, and dyslipidemia to assess the role of these variables in the associations. Tests for trend were performed using the Wald test for continuous trend variables. In secondary analyses, we stratified the analyses by potential effect modifiers (age, sex, region, household income, and BMI). Tests for interaction were performed using the Wald test for product terms. In sensitivity analyses, we further reduced confounding due to subclinical disease at baseline by conducting a 2-y and 5-y lag analyses after excluding deaths that occurred during the first 2 years (n = 116) and 5 years (n = 617) of follow-up, respectively. To examine the association between physical activity and prevalent health conditions, we estimated age- and sex-adjusted prevalence of type 2 diabetes, hypertension, and dyslipidemia by physical activity categories. We performed the F test for ANOVA to determine any difference in distributions by physical activity category. All statistical analyses were performed using SAS 9.4 (SAS Institute Inc.).

## Results

### Participant characteristics

During the follow-up of up to 12.4 years, 34,379 participants contributed a total of 315,226 person-years and 1,622 deaths. Mean duration of follow-up was 9.2 years and mean age at baseline was 47.9 years. Table 1 shows the baseline characteristics of study participants according to the categories of total aerobic physical activity. Compared to individuals with low levels of total aerobic physical activity, those with high levels were more likely to be male, younger, and have higher household income, good self-rated health, and higher levels of walking, muscle-strengthening, and flexibility physical activities. Similar patterns were observed by the levels of muscle-strengthening (Supplementary Table 1) and flexibility physical activities (Supplementary Table 2).

**Table 1:** Baseline characteristics according to categories of total aerobic physical activity^a^

Characteristics	Total aerobic physical activity (including walking; IPAQ guideline)^b^		
	Low	Moderate	High
	(n = 13,764)	(n = 11,622)	(n = 8,993)
	**N (Percentage** ^**c**^ **)**		
**Male**	5,236 (38.0)	4,774 (41.1)	4,694 (52.2)
**Age, years**			
19–49	7,384 (53.7)	6,494 (55.9)	5,036 (56.0)
50–59	2,586 (18.8)	2,030 (17.5)	1,918 (21.3)
60–69	2,115 (15.4)	1,822 (15.7)	1,386 (15.4)
≥ 70	1,679 (12.2)	1,276 (11.0)	653 (7.3)
**Region** ^**d**^			
Metropolitan	6,070 (44.1)	5,642 (48.6)	3,850 (42.8)
Urban	4,844 (35.2)	4,006 (34.5)	3,071 (34.2)
Rural	2,850 (20.7)	1,974 (17.0)	2,072 (23.0)
**Education attainment**			
Lower than high school	5,118 (37.2)	3,665 (31.5)	2,934 (32.6)
High school	4,548 (33.0)	3,992 (34.4)	3,605 (40.1)
College or higher	4,098 (29.8)	3,965 (34.1)	2,454 (27.3)
**Occupation**			
Nonphysical labor	2,899 (21.1)	2,852 (24.5)	1,647 (18.3)
Physical labor	5,201 (37.8)	3,972 (34.2)	4,648 (51.7)
Unemployed/homemakers/students/others	5,664 (41.2)	4,798 (41.3)	2,698 (30.0)
**Household income**			
Quartile 1	2,609 (19.0)	2,002 (17.2)	1,377 (15.3)
Quartile 2	3,568 (25.9)	2,828 (24.3)	2,323 (25.8)
Quartile 3	3,765 (27.4)	3,178 (27.3)	2,493 (27.7)
Quartile 4	3,611 (26.2)	3,440 (29.6)	2,678 (29.8)
Missing	211 (1.5)	174 (1.5)	122 (1.4)
**Marital status**			
Married	10,343 (75.2)	8,299 (71.4)	6,671 (74.2)
Never married	1,591 (11.6)	2,010 (17.3)	1,491 (16.6)
Divorced/separated/widowed	1,830 (13.3)	1,313 (11.3)	831 (9.2)
**Smoking status**			
Never	8,849 (64.3)	7,445 (64.1)	4,893 (54.4)
Former	2,015 (14.6)	1,886 (16.2)	1,788 (19.9)
Current	2,900 (21.1)	2,291 (19.7)	2,312 (25.7)
**Alcohol drinking**			
Nondrinkers	4,053 (29.5)	3,107 (26.7)	1,966 (21.9)
1 time/month	4,091 (29.7)	3,507 (30.2)	2,446 (27.2)
≥ 2 times/month	5,620 (40.8)	5,008 (43.1)	4,581 (50.9)
**Body Mass Index, kg/m** ^**2**^			
< 18.5	683 (5.0)	539 (4.6)	295 (3.3)
18.5–24.9	8,735 (63.5)	7,526 (64.8)	5,688 (63.3)
≥ 25.0	4,346 (31.6)	3,557 (30.6)	3,010 (33.5)
**Self-rated health**			
Good	4,196 (30.5)	4,492 (38.7)	4,086 (45.4)
Fair	6,708 (48.7)	4,996 (43.0)	3,402 (37.8)
Poor	2,860 (20.8)	2,134 (18.4)	1,505 (16.7)
**Walking, h/wk**			
0	4,399 (32.0)	247 (2.1)	430 (4.8)
0.1–2.4	6,859 (49.8)	2,285 (19.7)	1,357 (15.1)
2.5–4.9	1,696 (12.3)	4,193 (36.1)	1,651 (18.4)
5.0-9.9	610 (4.4)	3,382 (29.1)	1,918 (21.3)
≥ 10.0	200 (1.5)	1,515 (13.0)	3,637 (40.4)
**Muscle-strengthening physical activity, d/wk**			
0	11,565 (84.0)	8,438 (72.6)	5,318 (59.1)
1	754 (5.5)	761 (6.6)	580 (6.5)
2	629 (4.6)	786 (6.8)	689 (7.7)
3–4	507 (3.7)	930 (8.0)	1,339 (14.9)
≥ 5	309 (2.2)	707 (6.1)	1,067 (11.9)
**Flexibility physical activity, d/wk**			
0	7,945 (57.7)	5,001 (43.0)	3,216 (35.8)
1	1,550 (11.3)	1,043 (9.0)	630 (7.0)
2	1,486 (10.8)	1,440 (12.4)	891 (9.9)
3–4	1,593 (11.6)	2,031 (17.5)	1,933 (21.5)
≥ 5	1,190 (8.7)	2,107 (18.1)	2,323 (25.8)

Abbreviations:

IPAQ, International Physical Activity Questionnaire

^a^ When there were less than 1% of missing data, values were imputed with the most frequent categories: education attainment (0.15%, n = 50), marital status (0.29%, n = 101), smoking status (0.04%, n = 13), alcohol drinking (0.24%, n = 82), body mass index (0.27% n = 92), and self-reported health (0.12%, n = 42). Those with missing information on occupation status (0.53%, n = 181) were combined into the “unemployed/homemakers/students/others” category.

^b^ The “high” category of total aerobic activity included participants with ≥ 3 days of vigorous-intensity activity achieving total aerobic activity of ≥ 25 MET-h/wk; or ≥ 7 days of any combination of walking, moderate-intensity, or vigorous-intensity activities achieving total aerobic physical activity of ≥ 50 MET-h/wk. The “moderate” category included participants who satisfied at least one of the following 3 criteria: (1) ≥ 3 days of vigorous-intensity activity of at least 20 min/d; (2) ≥ 5 days of moderate-intensity activity and/or walking of at least 30 min/d; or (3) ≥ 5 days of any combination of walking, moderate-intensity, or vigorous-intensity activities achieving total aerobic physical activity of ≥ 10 MET-h/wk. The “low” category included participants who were not meeting criteria for “moderate” and “high” categories.

^c^ Values may not sum to 100% due to rounding.

^d^ Metropolitan includes Seoul capital city and 6 other metropolitan cities (Busan, Daegu, Incheon, Gwangju, Daejeon, and Ulsan). Urban and rural are defined by the legal distribution of submunicipal level divisions based on the size of area and population (urban; ‘Dong’, rural; ‘Eup/Myeon’).

### Prevalent health conditions

Table 2 shows the age- and sex-adjusted prevalence of types 2 diabetes, hypertension, and dyslipidemia at baseline according to physical activity categories. For each type of physical activity (total aerobic, moderate- to vigorous-intensity aerobic, muscle-strengthening, flexibility), participants with higher levels of activity were more likely to have a lower prevalence of dyslipidemia.

**Table 2:** Age- and sex-adjusted prevalence of type 2 diabetes, hypertension, and dyslipidemia at baseline according to physical activity categories

Prevalent conditions at baseline					
	N	Type 2 diabetes,^a^ %	Hypertension,^b^ %	Dyslipidemia,^c^ %	Number of prevalent health conditions^d^ (0–3), mean ± SE
**Total aerobic physical activity (including walking; IPAQ guideline)** ^**e**^					
Low	13,764	10.7	29.9	44.4	0.85 ± 0.007
Moderate	11,622	10.8	29.8	42.3	0.83 ± 0.007
High	8993	9.6	29.5	40.5	0.80 ± 0.008
*P* value^f^		0.003	0.76	< 0.001	< 0.001
**Moderate- to vigorous-intensity aerobic physical activity (MET-h/wk)** ^**g**^					
0	16,926	11.3	29.8	43.2	0.84 ± 0.006
0.1–4.9	1988	10.1	30.1	44.3	0.85 ± 0.017
5.0-9.9	2134	8.9	29.2	42.2	0.80 ± 0.017
10.0-19.9	3121	9.6	30.5	42.7	0.83 ± 0.014
20.0-49.9	5309	9.5	29.7	43.0	0.82 ± 0.011
≥ 50.0	4901	9.8	29.5	40.0	0.79 ± 0.011
*P* value^f^		< 0.001	0.89	< 0.001	0.001
**Muscle-strengthening physical activity (d/wk)**					
0	25,321	10.6	30.0	43.3	0.84 ± 0.005
1	2095	9.9	29.6	41.5	0.81 ± 0.017
2	2104	9.6	28.3	42.6	0.80 ± 0.017
3–4	2776	10.2	27.9	39.9	0.78 ± 0.015
≥ 5	2083	10.4	31.4	39.7	0.82 ± 0.017
*P* value^f^		0.52	0.02	< 0.001	< 0.001
**Flexibility physical activity (d/wk)**					
0	16,162	10.9	30.3	43.4	0.85 ± 0.006
1	3223	10.2	29.9	43.3	0.83 ± 0.014
2	3817	10.0	29.3	42.0	0.81 ± 0.012
3–4	5557	9.9	28.6	42.0	0.81 ± 0.010
≥ 5	5620	10.0	29.7	41.3	0.81 ± 0.010
*P* value^f^		0.08	0.12	0.03	0.001

Abbreviations: SE, Standard Error; IPAQ, International Physical Activity Questionnaire; MET, metabolic equivalent task

^a^ Type 2 diabetes was defined as those who reported a prior diagnosis of type 2 diabetes, were currently taking diabetic medication, or had measured fasting blood glucose concentrations ≥ 126 mg/dL or HbA1c ≥ 6.5% at baseline.

^b^ Hypertension was defined as having a prior diagnosis of hypertension, currently taking antihypertensive medication, or having a measured systolic blood pressure ≥ 140 mmHg or diastolic blood pressure ≥ 90 mmHg at baseline.

^c^ Dyslipidemia was defined as having a prior diagnosis of dyslipidemia, currently taking medication or on treatment, or having measured total cholesterol concentrations ≥ 240 mg/dL, triglyceride concentrations ≥ 200 mg/dL, LDL cholesterol concentrations ≥ 160 mg/dL, or HDL cholesterol concentrations ≤ 40 mg/dL.

^d^ Summed number of prevalent health conditions (type 2 diabetes, hypertension, and dyslipidemia)

^e^ The “high” category of total aerobic activity included participants with ≥ 3 days of vigorous-intensity activity achieving total aerobic activity of ≥ 25 MET-h/wk; or ≥ 7 days of any combination of walking, moderate-intensity, or vigorous-intensity activities achieving total aerobic physical activity of ≥ 50 MET-h/wk. The “moderate” category included participants who satisfied at least one of the following 3 criteria: (1) ≥ 3 days of vigorous-intensity activity of at least 20 min/d; (2) ≥ 5 days of moderate-intensity activity and/or walking of at least 30 min/d; or (3) ≥ 5 days of any combination of walking, moderate-intensity, or vigorous-intensity activities achieving total aerobic physical activity of ≥ 10 MET-h/wk. The “low” category included participants who were not meeting criteria for “moderate” and “high” categories.

^f^*P* value was estimated by *F* test for ANOVA.

^g^ Walking was not included when estimating MET-h/wk.

### Physical activity type and all-cause mortality

Table 3 presents the associations of aerobic, muscle-strengthening, and flexibility physical activities with all-cause mortality. Compared with participants who did not perform any moderate- to vigorous-intensity aerobic physical activity, those with higher levels of moderate- to vigorous-intensity aerobic activity were associated with lower all-cause mortality risk (≥ 50.0 vs. 0 MET-h/wk: HR = 0.82; 95% CI = 0.70–0.95; *P*-trend < 0.001). The associations remained statistically significant after additional adjustment for BMI (HR = 0.82, 95% CI = 0.71–0.95; *P*-trend < 0.001) and after adjustment for prevalent conditions (HR = 0.81, 95% CI = 0.70–0.95; *P*-trend < 0.001). In all models, the inverse association between moderate- to vigorous-intensity aerobic activity and all-cause mortality was statistically significant at ≥ 5.0 MET-h/wk (5.0-9.9 vs. 0 MET-h/wk: HR = 0.64; 95% CI = 0.48–0.85 in Model 1). Including walking, the statistically significant inverse association was observed at ≥ 10.0 MET-h/wk (HR = 0.81; 95% CI = 0.68–0.96; *P*-trend = 0.02; Supplementary Table 3). Compared with “low” level of total aerobic activity (including walking) based on the IPAQ guideline, the “high” level was also inversely associated with all-cause mortality but the association was marginally significant (HR = 0.89, 95% CI = 0.78–1.01; *P*-trend = 0.07). Performing muscle-strengthening physical activity for at least 1 d/wk was also inversely associated with all-cause mortality (1 vs. 0 d/wk: HR = 0.67; 95% CI = 0.48–0.94; *P*-trend = 0.01). Performing flexibility physical activity for at least 1 d/wk was statistically significantly associated with a lower risk of all-cause mortality (1 vs. 0 d/wk: HR = 0.70; 95% CI = 0.53–0.91; *P*-trend < 0.001). For each activity type, the inverse association did not change after additional adjustment for BMI or prevalent conditions.

**Table 3:** Associations between three physical activity types (aerobic, muscle-strengthening, flexibility) and all-cause mortality

	Death/person-year^a^	Model 1^b^ HR (95% CIs)	Model 2^c^ HR (95% CIs)	Model 3^d^ HR (95% CIs)
**Total aerobic physical activity (including walking; IPAQ guideline)** ^**e**^				
Low	697/123,540	1.00 (ref)	1.00 (ref)	1.00 (ref)
Moderate	556/106,556	0.96 (0.85–1.07)	0.95 (0.85–1.07)	0.95 (0.85–1.06)
High	369/85,129	0.89 (0.78–1.01)	0.88 (0.78–1.01)	0.88 (0.78–1.01)
*P*-trend^f^		0.07	0.06	0.06
**Moderate- to vigorous-intensity aerobic physical activity (MET-h/wk)** ^**g**^				
0	1058/152,634	1.00 (ref)	1.00 (ref)	1.00 (ref)
0.1–4.9	63/18,353	0.81 (0.63–1.04)	0.81 (0.63–1.05)	0.80 (0.62–1.04)
5.0-9.9	52/19,756	0.64 (0.48–0.85)	0.65 (0.49–0.86)	0.65 (0.49–0.86)
10.0-19.9	80/28,657	0.69 (0.55–0.87)	0.69 (0.54–0.87)	0.69 (0.55–0.87)
20.0-49.9	153/49,192	0.76 (0.63–0.90)	0.76 (0.64–0.91)	0.76 (0.64–0.90)
≥ 50.0	216/46,633	0.82 (0.70–0.95)	0.82 (0.71–0.95)	0.81 (0.70–0.95)
*P*-trend^f^		< 0.001	< 0.001	< 0.001
**Muscle-strengthening physical activity (d/wk)**				
0	1354/232,365	1.00 (ref)	1.00 (ref)	1.00 (ref)
1	37/19,096	0.67 (0.48–0.94)	0.68 (0.49–0.95)	0.67 (0.48–0.94)
2	50/19,110	0.82 (0.62–1.08)	0.81 (0.61–1.08)	0.82 (0.62–1.08)
3–4	75/25,317	0.81 (0.64–1.04)	0.81 (0.63–1.03)	0.80 (0.63–1.02)
≥ 5	106/19,337	0.83 (0.68–1.02)	0.84 (0.69–1.03)	0.82 (0.67–1.01)
*P*-trend^f^		0.01	0.01	0.01
**Flexibility physical activity (d/wk)**				
0	1,069/148,736	1.00 (ref)	1.00 (ref)	1.00 (ref)
1	62/29,151	0.70 (0.53–0.91)	0.69 (0.53–0.91)	0.69 (0.53–0.91)
2	81/34,676	0.72 (0.58–0.91)	0.72 (0.58–0.90)	0.73 (0.58–0.91)
3–4	149/50,533	0.85 (0.71–1.01)	0.84 (0.71–1.01)	0.86 (0.72–1.02)
≥ 5	261/52,129	0.80 (0.70–0.92)	0.81 (0.70–0.93)	0.80 (0.70–0.93)
*P*-trend^f^		< 0.001	< 0.001	< 0.001

Abbreviation: CIs, Confidence Intervals; HR, Hazard Ratio; IPAQ, International Physical Activity Questionnaire; MET, metabolic equivalent task

^a^ Person-years may not sum to the total person-years of 315,226 due to rounding.

^b^ Model 1 includes sex (male, female), region (metropolitan, urban, rural), education (less than high school, high school, college or higher), occupation (nonphysical labor, physical labor, unemployed/homemakers/students/others), household income level (quartiles 1,2,3,4, missing), marital status (married, never married, divorced/separated/widowed), smoking status (never, former, current), alcohol drinking (nondrinkers, 1 time/month, ≥ 2 times/month), and self-rated health (good, fair, poor).

^c^ Model 2 includes all variables in Model 1 plus body mass index (< 18.5, 18.5–24.9, ≥ 25.0 kg/m^2^).

^d^ Model 3 includes all variables in Model 1 plus prevalence of type 2 diabetes (present, absent), hypertension (present, absent), and dyslipidemia (present, absent).

^e^ The “high” category of total aerobic activity included participants with ≥ 3 days of vigorous-intensity activity achieving total aerobic activity of ≥ 25 MET-h/wk; or ≥ 7 days of any combination of walking, moderate-intensity, or vigorous-intensity activities achieving total aerobic physical activity of ≥ 50 MET-h/wk. The “moderate” category included participants who satisfied at least one of the following 3 criteria: (1) ≥ 3 days of vigorous-intensity activity of at least 20 min/d; (2) ≥ 5 days of moderate-intensity activity and/or walking of at least 30 min/d; or (3) ≥ 5 days of any combination of walking, moderate-intensity, or vigorous-intensity activities achieving total aerobic physical activity of ≥ 10 MET-h/wk. The “low” category included participants who were not meeting criteria for “moderate” and “high” categories.

^f^*P*-trend was estimated using the Wald test for continuous trend variable.

^g^ Walking was not included when estimating MET-h/wk.

### Physical activity type and cause-specific mortality

Table 4 presents the associations with cause-specific mortality. Moderate- to vigorous-intensity aerobic physical activity was inversely associated with both cancer (10.0-19.9 vs. 0 MET-h/wk: HR = 0.66; 95% CI = 0.45–0.98; *P*-trend = 0.15) and cardiovascular mortality (≥ 50.0 vs. 0 MET-h/wk: HR = 0.55; 95% CI = 0.37–0.80; *P*-trend < 0.001). Including walking, total aerobic physical activity was inversely associated with cardiovascular mortality (high vs. low: HR = 0.56, 95% CI = 0.40–0.77; *P*-trend < 0.001) but was not associated with cancer mortality (HR = 0.96, 95% CI = 0.77–1.19; *P*-trend = 0.81). Flexibility physical activity was also inversely associated with cardiovascular mortality (3–4 vs. 0 d/wk: HR = 0.61; 95% CI = 0.39–0.96; *P*-trend = 0.02) but was not associated with cancer mortality (≥ 5 vs. 0 d/wk: HR = 0.87; 95% CI = 0.69–1.09; *P*-trend = 0.27). Muscle-strengthening activity (≥ 5 vs. 0 d/wk) was not associated with cancer (HR = 0.90; 95% CI = 0.65–1.26; *P*-trend = 0.76) or cardiovascular mortality (HR = 0.94; 95% CI = 0.60–1.47; *P*-trend = 0.45).

**Table 4:** Associations between three physical activity types (aerobic, muscle-strengthening, flexibility) and cause-specific mortality (cancer- and cardiovascular disease-specific mortality)

	Cancer mortality		Cardiovascular mortality	
	Death/person-year ^a^	HR (95% CIs)^b^	Death/person-year^a^	HR (95% CIs)^b^
**Total aerobic physical activity (including walking; IPAQ guideline)** ^**c**^				
Low	229/123,540	1.00 (ref)	168/123,540	1.00 (ref)
Moderate	207/106,556	1.07 (0.88–1.29)	118/106,556	0.87 (0.68–1.10)
High	138/85,129	0.96 (0.77–1.19)	52/85,129	0.56 (0.40–0.77)
*P*-trend^d^		0.81		< 0.001
**Moderate- to vigorous-intensity aerobic physical activity (MET-h/wk)** ^**e**^				
0	357/152,634	1.00 (ref)	248/152,634	1.00 (ref)
0.1–4.9	23/18,353	0.89 (0.58–1.36)	11/18,353	0.65 (0.35–1.19)
5.0-9.9	21/19,756	0.77 (0.49–1.19)	-^f^/19,756	0.45 (0.22–0.91)
10.0-19.9	27/28,657	0.66 (0.45–0.98)	16/28,657	0.64 (0.38–1.06)
20.0-49.9	57/49,192	0.78 (0.59–1.04)	23/49,192	0.55 (0.36–0.85)
≥ 50.0	89/46,633	0.96 (0.75–1.22)	32/46,633	0.55 (0.37–0.80)
*P*-trend^d^		0.15		< 0.001
**Muscle-strengthening physical activity (d/wk)**				
0	452/232,365	1.00 (ref)	292/232,365	1.00 (ref)
1–2	45/38,206	1.08 (0.78–1.49)	14/38,206	0.73 (0.42–1.27)
3–4	36/25,317	1.07 (0.75–1.51)	11/25,317	0.74 (0.40–1.36)
≥ 5	41/19,337	0.90 (0.65–1.26)	21/19,337	0.94 (0.60–1.47)
*P*-trend^d^		0.76		0.45
**Flexibility physical activity (d/wk)**				
0	351/148,736	1.00 (ref)	245/148,736	1.00 (ref)
1–2	59/63,827	0.85 (0.64–1.13)	21/63,827	0.52 (0.33–0.82)
3–4	65/50,533	1.00 (0.76–1.32)	21/50,533	0.61 (0.39–0.96)
≥ 5	99/52,129	0.87 (0.69–1.09)	51/52,129	0.75 (0.55–1.03)
*P*-trend^d^		0.27		0.02

Abbreviation: CIs, Confidence Intervals; HR, Hazard Ratio; IPAQ, International Physical Activity Questionnaire; MET, metabolic equivalent task

^a^ Person-years may not sum to the total person-years of 315,226 due to rounding.

^b^ Adjusted for sex (male, female), region (metropolitan, urban, rural), education (< high school, high school, college or higher), occupation (nonphysical labor, physical labor, unemployed/homemakers/students/others), household income level (quartile 1,2,3,4, missing), marital status (married, never married, divorced/separated/widowed), smoking status (never, former, current), alcohol drinking (nondrinkers, 1 time/month, ≥ 2 times/month), and self-rated health (good, fair, poor)

^c^ The “high” category of total aerobic activity included participants with ≥ 3 days of vigorous-intensity activity achieving total aerobic activity of ≥ 25 MET-h/wk; or ≥ 7 days of any combination of walking, moderate-intensity, or vigorous-intensity activities achieving total aerobic physical activity of ≥ 50 MET-h/wk. The “moderate” category included participants who satisfied at least one of the following 3 criteria: (1) ≥ 3 days of vigorous-intensity activity of at least 20 min/d; (2) ≥ 5 days of moderate-intensity activity and/or walking of at least 30 min/d; or (3) ≥ 5 days of any combination of walking, moderate-intensity, or vigorous-intensity activities achieving total aerobic physical activity of ≥ 10 MET-h/wk. The “low” category included participants who were not meeting criteria for “moderate” and “high” categories.

^d^*P*-trend was estimated using the Wald test for continuous trend variable.

^e^ Walking was not included when estimating MET-h/wk.

^f^ Estimates for death count < 10 is not disclosed.

### Physical activity guidelines and mortality

Table 5 presents the joint associations of meeting aerobic and muscle-strengthening physical activity guidelines with all-cause and cause-specific mortality. Compared to participants meeting the highest guidelines for both total aerobic (“high” based on the IPAQ guideline) and muscle-strengthening (≥ 2 d/wk) activities, those not meeting any physical activity guideline (“low” total aerobic and < 2 d/wk muscle-strengthening) were statistically significantly associated with higher risks of all-cause (HR = 1.28; 95% CI = 1.02–1.62) and cardiovascular mortality (HR = 2.04; 95% CI = 1.08-3.88). Using the WHO guidelines for moderate- to vigorous-intensity aerobic and muscle-strengthening activities, not meeting any guideline was associated with a 34% higher risk of all-cause mortality (95% CI = 1.09–1.64) and a 68% higher risk of cardiovascular mortality (95% CI = 1.00-2.82), whereas those satisfying at least one guideline were not statistically significantly associated with higher all-cause and cause-specific mortality. None of the interactions between aerobic and muscle-strengthening guidelines was statistically significant (*P*-interaction ≥ 0.27).

**Table 5:** Joint associations of meeting aerobic and muscle-strengthening physical activity guidelines with all-cause and cause-specific mortality

		All-cause mortality		Cancer mortality		Cardiovascular mortality	
Total aerobic activity (including walking; IPAQ guideline)^c^	Muscle-strengthening activity	Death/ person-year^a^	HR (95% CIs)^b^	Death/ person-year^a^	HR (95% CIs)^b^	Death/ person-year^a^	HR (95% CIs)^b^
**Low**	**< 2 d/wk**	640/110,496	1.28 (1.02–1.62)	205/110,496	1.07 (0.74–1.55)	155/110,496	2.04 (1.08–3.88)
**Low**	**≥ 2 d/wk**	57/13,044	1.04 (0.74–1.46)	24/13,044	1.06 (0.63–1.78)	13/13,044	1.94 (0.85–4.42)
**Moderate**	**< 2 d/wk**	469/84,555	1.23 (0.97–1.55)	167/84,555	1.14 (0.79–1.65)	100/84,555	1.75 (0.91–3.35)
**Moderate**	**≥ 2 d/wk**	87/22,001	1.07 (0.80–1.44)	40/22,001	1.15 (0.73–1.81)	18/22,001	1.83 (0.86–3.90)
**High**	**< 2 d/wk**	282/56,411	1.16 (0.91–1.47)	101/56,411	1.03 (0.70–1.52)	42/56,411	1.17 (0.59–2.34)
**High**	**≥ 2 d/wk**	87/28,719	1.00 (ref)	37/28,719	1.00 (ref)	10/28,719	1.00 (ref)
*P*-interaction^f^		0.92		0.99		0.89	
**Moderate- to vigorous-intensity aerobic physical activity (WHO guideline)** ^**d**^	**Muscle-strengthening activity**	**Death/** **person-year** ^**a**^	**HR (95% CIs)** ^**b**^	**Death/** **person-year** ^**a**^	**HR (95% CIs)** ^**b**^	**Death/** **person-year** ^**a**^	**HR (95% CIs)** ^**b**^
**< 10.0 MET-hr/wk**	**< 2 d/wk**	957/135,502	1.34 (1.09–1.64)	316/135,502	1.06 (0.78–1.45)	224/135,502	1.68 (1.00-2.82)
**< 10.0 MET-hr/wk**	**≥ 2 d/wk**	101/17,132	1.10 (0.84–1.45)	41/17,132	0.98 (0.65–1.48)	24/17,132	1.72 (0.92–3.24)
**10.0-19.9 MET-hr/wk**	**< 2 d/wk**	100/31,056	1.01 (0.77–1.32)	37/31,056	0.91 (0.60–1.40)	18/31,056	1.03 (0.52–2.02)
**10.0-19.9 MET-hr/wk**	**≥ 2 d/wk**	15/7,053	0.67 (0.39–1.15)	-^e^/7,053	0.69 (0.32–1.53)	-^e^/7,053	0.32 (0.05–2.31)
**≥ 20.0 MET-hr/wk**	**< 2 d/wk**	334/84,903	1.01 (0.81–1.25)	120/84,903	0.83 (0.60–1.16)	55/84,903	0.95 (0.54–1.65)
**≥ 20.0 MET-hr/wk**	**≥ 2 d/wk**	115/39,579	1.00 (ref)	53/39,579	1.00 (ref)	16/39,579	1.00 (ref)
*P*-interaction^f^		0.27		0.41		0.50	

Abbreviation: CIs, Confidence Intervals; HR, Hazard Ratio; IPAQ, International Physical Activity Questionnaire; MET, metabolic equivalent task

^a^ Person-years may not sum to the total person-years of 315,226 due to rounding.

^b^ Adjusted for sex (male, female), region (metropolitan, urban, rural), education (< high school, high school, college or higher), occupation (nonphysical labor, physical labor, unemployed/homemakers/students/others), household income level (quartile 1,2,3,4, missing), marital status (married, never married, divorced/separated/widowed), smoking status (never, former, current), alcohol drinking (nondrinkers, 1 time/month, ≥ 2 times/month), and self-rated health (good, fair, poor)

^c^ The “high” category of total aerobic activity included participants with ≥ 3 days of vigorous-intensity activity achieving total aerobic activity of ≥ 25 MET-h/wk; or ≥ 7 days of any combination of walking, moderate-intensity, or vigorous-intensity activities achieving total aerobic physical activity of ≥ 50 MET-h/wk. The “moderate” category included participants who satisfied at least one of the following 3 criteria: (1) ≥ 3 days of vigorous-intensity activity of at least 20 min/d; (2) ≥ 5 days of moderate-intensity activity and/or walking of at least 30 min/d; or (3) ≥ 5 days of any combination of walking, moderate-intensity, or vigorous-intensity activities achieving total aerobic physical activity of ≥ 10 MET-h/wk. The “low” category included participants who were not meeting criteria for “moderate” and “high” categories.

^d^ Walking was not included when estimating MET-h/wk.

^e^ Estimates for death count < 10 is not disclosed.

^f^*P*-interaction was estimated using the Wald test for product terms between aerobic physical activity (low, moderate, high) and muscle-strengthening physical activity (< 2, ≥ 2).

In stratified analysis, similar associations were consistently observed in subgroups defined by sex, age, region, income, and BMI. None of the interactions was statistically significant (Supplemental Tables 4–6). Results were also similar in 2-y (Supplementary Tables 7–8) and 5-y lag analyses (Supplementary Tables 9–10).

## Discussion

In a nationally representative sample of Korean adults, we found that aerobic (both including and excluding walking), muscle-strengthening, and flexibility physical activities were all associated with lower risks of all-cause mortality. Engaging in aerobic and flexibility physical activities was also associated with lower risk of cardiovascular mortality. Compared with participants meeting some guidelines for moderate- to vigorous-intensity aerobic and muscle-strengthening activities, not meeting any recommended guideline was associated with a 34% higher all-cause mortality and a 68% higher cardiovascular mortality. Our data support the inverse associations of aerobic, muscle-strengthening, and flexibility physical activities with mortality.

Among the three physical activity types that we examined, the most pronounced association was observed with aerobic physical activity. We observed the inverse associations of aerobic physical activity with the risks of all-cause, cancer, and cardiovascular mortality. The inverse associations with all-cause and cardiovascular mortality were also observed at the levels lower than those recommended by the WHO physical activity guideline [15]. Our finding is consistent with those from previous studies that reported even a minimum amount of aerobic physical activity is better than none for health benefits [28, 29]. In our study, we also observed a stronger inverse association with cardiovascular mortality than with cancer mortality. Several potential mechanisms may explain the association between aerobic activity and mortality. Aerobic physical activity is associated with lower blood pressure [30, 31], reduced inflammation [32–34], insulin sensitivity [35, 36], and improved lipid profile [34, 37] and endothelial function [38, 39], leading to lower risks of atherosclerosis and thrombosis. Aerobic physical activity is also associated with lower body fat [2, 3], while excess body fat is an important risk factor for cardiovascular disease [40, 41] and certain cancer sites [42, 43]. In our analysis, the inverse association between aerobic activity and mortality persisted after additional adjustment for BMI, suggesting that BMI-independent pathways (e.g., immune function) may also exist. Studies have also shown that aerobic physical activity may lower the risks of certain cancer sites (bladder, breast, colon, endometrial, esophageal adenocarcinoma, gastric cardia, and renal cancers)[44–49] and improve cancer survival [44, 50–52] by regulating tumor growth and progression and alleviating adverse events related to cancer treatment. In our study, we observed no additional mortality risk reduction at the highest level of physical activity. Similar to our finding, previous pooled analyses also consistently reported no significant additional benefit beyond the levels recommended by the physical activity guidelines [53, 54]. The possible explanation for this finding is that there are typically fewer participants at the highest level of physical activity and thus the results are subject to high random variabilities. It is also possible that extremely high levels of physical activity may have some negative health effects such as injuries [55, 56], fractures [57, 58], and poor sleep quality [59, 60].

Our finding of inverse associations between flexibility physical activity and all-cause and cardiovascular mortality is noteworthy in that few studies to date have examined the associations. Consistently, a previous follow-up study from the U.S. reported that stretching was independently associated with a lower risk of all-cause mortality while adjusting for 14 other types of exercise, such as soccer, tennis, and golf [61]. Health benefits of flexibility physical activity may include reducing risks of injuries [16–18] and chronic myofascial pain syndrome [62]. Increased body balance and flexibility may also reduce the risk of falls and fractures [63, 64] and help recovery after injuries [65]. Flexibility training may also improve immune function [66, 67], metabolism [68–70], and circulatory health [71]. Through deep breathing and gentle movements, flexibility exercise is also associated with reduced stress and anxiety [66, 72], contributing to improved blood pressure recovery from stress [68].

In our study, we observed a marginally significant association between high levels of muscle-strengthening physical activity and lower mortality. The limited statistical significance in our study may be due to the limited sample size. Our study also used muscle-strengthening physical activity data that were reported in the number of days per week, without information collected on the average duration per day. If the frequency (number of days) did not correlate with the total duration, misclassification may have occurred and attenuated the association. In addition, if the duration per day was very short for most participants who performed muscle-strengthening activity, the risk reduction may not have been large enough. Further, the survey did not collect information on the specific muscle-strengthening activity performed (e.g., push-up, sit-up). Some studies [73–75] suggest the potential differences in biological mechanisms among different muscle-strengthening activities. In a meta-analysis that examined the associations of resistance training [76], the magnitude of blood pressure reduction varied by the type of muscle contraction, whether dynamic (both the length and the tension of the muscles change) or static/isometric (the length does not change). In an experimental study [77], the associations with blood pressure reduction also varied by the intensity of resistance exercise. It is also possible that physical fitness [78] or the amount of muscle [79–81] may be more important than the engagement level of muscle-strengthening activity. Further studies are needed to clarify the associations related to detailed activity and intensity types of muscle-strengthening physical activity.

We acknowledge several limitations of our study. We used self-reported physical activity data which are subject to measurement error. However, in our prospective analysis, the measurement error is likely to be non-differential and only underestimated the associations between physical activity and mortality. Further, the Korean version of the IPAQ questionnaire has been previously validated against accelerometer measurements [24]. Our physical activity data were also collected once at baseline and thus we were not able to account for changing patterns of physical activity during the follow-up. Finally, given the limited follow-up time, we cannot fully exclude the possibility of confounding by subclinical diseases at baseline. However, our findings were shown robust after excluding deaths that occurred during the first 5 years of follow-up.

Despite the limitations, our study has several strengths. Our study used a prospective study design and thereby reduced possible recall bias. Our results were also shown robust after adjustment of potential confounders, including demographic, socioeconomic, and lifestyle factors. We also considered three different types of physical activity, including less-studied activity type, namely flexibility activity. While few studies examined the associations in Asian populations, we examined the associations in Korean adults. Our findings may provide additional insights into the health benefits of physical activity in Asian populations. Further, by including a nationally representative sample of Korean adults, we also increased the generalizability of our study finding.

In conclusion, aerobic, muscle-strengthening, and flexibility physical activities were associated with reduced risks of all-cause mortality in Korean adults. Additionally, aerobic and flexibility activities were also associated with lower risks of cardiovascular mortality. Although the current physical activity guidelines heavily focus on aerobic and muscle-strengthening physical activities, preventive strategies may also promote flexibility physical activity to reduce premature deaths in adults.

## Electronic supplementary material

Below is the link to the electronic supplementary material.

Supplementary Material 1

## Data Availability

The datasets used and/or analyzed during the current study are publicly available at the Korea Disease Control and Prevention Agency repository (https://knhanes.kdca.go.kr). Linkage with mortality data is available after approval from the KDCA.
