# Supplementary material for: Aerobic, muscle-strengthening, and flexibility physical activity and risks of all-cause and cause-specific mortality: a population-based prospective cohort of Korean adults
Source: BMC Public Health. 2023 Jun 14;23:1148. doi: 10.1186/s12889-023-15969-1 (PMC10268385; doi:10.1186/s12889-023-15969-1)
Supplement: Supplementary file 1 — Supplementary Material 1 [file 12889_2023_15969_MOESM1_ESM.docx]

**ONLINE SUPPLEMENTARY MATERIAL**

**Aerobic, muscle-strengthening, and flexibility physical activity and risks of all-cause and cause-specific mortality: a population-based prospective cohort of Korean adults**

**Yoonkyoung Cho, et al.**

**Supplementary Figure 1. Participant flowchart**

Final analytic dataset:

34,379 KNHANES 2007-2013 participants with mortality data

Excluded participants who reported to have lied down all day due to health problems

(n=160)

Excluded participants who died during the first year of follow-up (n=94)

Excluded participants who had an unreasonable range of aerobic physical activity

(n=66)

Excluded participants who had missing information on any type of physical activity

(n=1,217)

Excluded participants who had a prior diagnosis of cancer or cardiovascular disease at baseline

(n=2,800)

Excluded participants who were pregnant at baseline

(n=227)

38,943 KNHANES 2007-2013 participants

At age 20-79 years

Excluded participants who did not agree to mortality data linkage

(n=1)

38,944 KNHANES 2007-2013 participants

At age 20-79 years

**Supplementary Table 1. Baseline characteristics according to levels of muscle-strengthening physical activity ^a^**

| **Characteristics** | **Muscle-strengthening physical activity (d/wk)** | | | | | |
| --- | --- | --- | --- | --- | --- | --- |
|  | **0** | | **1** | **2** | **3-4** | **≥5**  **(n=2,083)** |
|  | **(n=25,321)** | | **(n=2,095)** | **(n=2,104)** | **(n=2,776)** |  |
|  | **N (Percentage^b^)** | | | | | |
| **Male** | 9,028 (35.7) | | 1,250 (59.7) | 1,252 (59.5) | 1,728 (62.3) | 1,446 (69.4) |
| **Age, years** |  | |  |  |  |  |
| 19-49 | 13,553 (53.5) | | 1,436 (68.5) | 1,323 (62.9) | 1,644 (59.2) | 958 (46.0) |
| 50-59 | 4,636 (18.3) | | 392 (18.7) | 453 (21.5) | 611 (22.0) | 442 (21.2) |
| 60-69 | 4,045 (16.0) | | 198 (9.5) | 236 (11.2) | 391 (14.1) | 453 (21.8) |
| ≥70 | 3,087 (12.2) | | 69 (3.3) | 92 (4.4) | 130 (4.7) | 230 (11.0) |
| **Region^c^** |  | |  |  |  |  |
| Metropolitan | 11,081 (43.8) | | 1,021 (48.7) | 1,047 (49.8) | 1,393 (50.2) | 1,020 (49.0) |
| Urban | 8,622 (34.1) | | 787 (37.6) | 742 (35.3) | 1,012 (36.5) | 758 (36.4) |
| Rural | 5,618 (22.2) | | 287 (13.7) | 315 (15.0) | 371 (13.4) | 305 (14.6) |
| **Education attainment** |  | |  |  |  |  |
| Lower than high school | 9,710 (38.4) | | 373 (17.8) | 441 (21.0) | 587 (21.2) | 606 (29.1) |
| High school | 8,374 (33.1) | | 835 (39.9) | 869 (41.3) | 1,169 (42.1) | 898 (43.1) |
| College or higher | 7,237 (28.6) | | 887 (42.3) | 794 (37.7) | 1,020 (36.7) | 579 (27.8) |
| **Occupation** |  | |  |  |  |  |
| Nonphysical labor | 5,001 (19.8) | | 620 (29.6) | 594 (28.2) | 770 (27.7) | 413 (19.8) |
| Physical labor | 10,306 (40.7) | | 821 (39.2) | 792 (37.6) | 1,064 (38.3) | 838 (40.2) |
| Unemployed/homemakers/  students/others | 10,014 (39.6) | | 654 (31.2) | 718 (34.1) | 942 (33.9) | 832 (39.9) |
| **Household income** |  | |  |  |  |  |
| Quartile 1 | 4,933 (19.5) | | 194 (9.3) | 225 (10.7) | 282 (10.2) | 354 (17.0) |
| Quartile 2 | 6,511 (25.7) | | 536 (25.6) | 496 (23.6) | 656 (23.6) | 520 (25.0) |
| Quartile 3 | 6,871 (27.1) | | 612 (29.2) | 604 (28.7) | 776 (28.0) | 573 (27.5) |
| Quartile 4 | 6,624 (26.2) | | 732 (34.9) | 747 (35.5) | 1,023 (36.9) | 603 (29.0) |
| Missing | 382 (1.5) | | 21 (1.0) | 32 (1.5) | 39 (1.4) | 33 (1.6) |
| **Marital status** |  | |  |  |  |  |
| Married | 18,651 (73.7) | | 1,516 (72.4) | 1,560 (74.1) | 2,049 (73.8) | 1,537 (73.8) |
| Never married | 3,351 (13.2) | | 446 (21.3) | 409 (19.4) | 536 (19.3) | 350 (16.8) |
| Divorced/separated/widowed | 3,319 (13.1) | | 133 (6.4) | 135 (6.4) | 191 (6.9) | 196 (9.4) |
| **Smoking status** |  | |  |  |  |  |
| Never | 16,635 (65.7) | | 1,120 (53.5) | 1,102 (52.4) | 1,395 (50.3) | 935 (44.9) |
| Former | 3,550 (14.0) | | 383 (18.3) | 455 (21.6) | 689 (24.8) | 612 (29.4) |
| Current | 5,136 (20.3) | | 592 (28.3) | 547 (26.0) | 692 (24.9) | 536 (25.7) |
| **Alcohol drinking** |  | |  |  |  |  |
| Nondrinkers | 7,517 (29.7) | | 346 (16.5) | 370 (17.6) | 479 (17.3) | 414 (19.9) |
| 1 time/month | 7,607 (30.0) | | 558 (26.6) | 565 (26.9) | 769 (27.7) | 545 (26.2) |
| ≥2 times/month | 10,197 (40.3) | | 1,191 (56.9) | 1,169 (55.6) | 1,528 (55.0) | 1,124 (54.0) |
| **Body Mass Index, kg/m^2^** |  | |  |  |  |  |
| <18.5 | 1,249 (4.9) | | 85 (4.1) | 68 (3.2) | 66 (2.4) | 49 (2.4) |
| 18.5-24.9 | 16,107 (63.6) | | 1,322 (63.1) | 1,391 (66.1) | 1,797 (64.7) | 1,332 (64.0) |
| ≥25.0 | 7,965 (31.5) | | 688 (32.8) | 645 (30.7) | 913 (32.9) | 702 (33.7) |
| **Self-rated health** |  | |  |  |  |  |
| Good | 8,539 (33.7) | | 895 (42.7) | 893 (42.4) | 1,321 (47.6) | 1,126 (54.1) |
| Fair | 11,353 (44.8) | | 944 (45.1) | 958 (45.5) | 1,145 (41.3) | 706 (33.9) |
| Poor | 5,429 (21.4) | | 256 (12.2) | 253 (12.0) | 310 (11.2) | 251 (12.1) |
| **Walking, h/wk** |  | |  |  |  |  |
| 0 | 4,487 (17.7) | | 139 (6.6) | 136 (6.5) | 152 (5.5) | 162 (7.8) |
| 0.1-2.4 | 7,982 (31.5) | | 777 (37.1) | 697 (33.1) | 717 (25.8) | 328 (15.8) |
| 2.5-4.9 | 5,315 (21.0) | | 463 (22.1) | 553 (26.3) | 723 (26.0) | 486 (23.3) |
| 5.0-9.9 | 3,867 (15.3) | | 367 (17.5) | 415 (19.7) | 659 (23.7) | 602 (28.9) |
| ≥10.0 | 3,670 (14.5) | | 349 (16.7) | 303 (14.4) | 525 (18.9) | 505 (24.2) |
| **Total aerobic physical activity (IPAQ guideline)** | | | |  |  |  |
| Low | 11,565 (45.7) | | 754 (36.0) | 629 (29.9) | 507 (18.3) | 309 (14.8) |
| Moderate | 8,438 (33.3) | | 761 (36.3) | 786 (37.4) | 930 (33.5) | 707 (33.9) |
| High | 5,318 (21.0) | | 580 (27.7) | 689 (32.8) | 1,339 (48.2) | 1,067 (51.2) |
| **Moderate- to vigorous-intensity aerobic physical activity, MET-h/wk** | | | | | |  |
| 0 | 14,407 (56.9) | | 611 (29.2) | 596 (28.3) | 656 (23.6) | 656 (31.5) |
| 0.1-4.9 | 1,476 (5.8) | | 204 (9.7) | 137 (6.5) | 111 (4.0) | 60 (2.9) |
| 5.0-9.9 | 1,441 (5.7) | | 245 (11.7) | 177 (8.4) | 182 (6.6) | 89 (4.3) |
| 10.0-19.9 | 1,973 (7.8) | | 299 (14.3) | 302 (14.4) | 377 (13.6) | 170 (8.2) |
| 20.0-49.9 | 3,020 (11.9) | | 441 (21.1) | 566 (26.9) | 831 (29.9) | 451 (21.7) |
| ≥50.0 | 3,004 (11.9) | | 295 (14.1) | 326 (15.5) | 619 (22.3) | 657 (31.5) |
| **Flexibility physical activity, d/wk** | |  |  |  |  |  |
| 0 | 15,212 (60.1) | | 257 (12.3) | 219 (10.4) | 256 (9.2) | 218 (10.5) |
| 1 | 2,167 (8.6) | | 770 (36.8) | 173 (8.2) | 83 (3.0) | 30 (1.4) |
| 2 | 2,317 (9.2) | | 431 (20.6) | 802 (38.1) | 219 (7.9) | 48 (2.3) |
| 3-4 | 2,787 (11.0) | | 406 (19.4) | 586 (27.9) | 1,618 (58.3) | 160 (7.7) |
| ≥5 | 2,838 (11.2) | | 231 (11.0) | 324 (15.4) | 600 (21.6) | 1,627 (78.1) |

Abbreviation: IPAQ, International Physical Activity Questionnaire; MET, metabolic equivalent task

^a^ When there were less than 1% of missing data, values were imputed with the most frequent categories: education attainment (0.15%, n=50), marital status (0.29%, n=101), smoking status (0.04%, n=13), alcohol drinking (0.24%, n=82), body mass index (0.27% n=92), and self-reported health (0.12%, n=42). Those with missing information on occupation status (0.53%, n=181) and those who reported “unemployed” were combined as the “unemployed/homemakers/students/others” category.

^b^ Values may not sum up to 100% due to rounding.

^c^ Metropolitan includes Seoul capital city and 6 other metropolitan cities (Busan, Daegu, Incheon, Gwangju, Daejeon, and Ulsan). Urban and rural are defined by the legal distribution of submunicipal level divisions based on the size of area and population (urban; ‘Dong’, rural; ‘Eup/Myeon’).

**Supplementary Table 2. Baseline characteristics according to levels of flexibility physical activity^a^**

| **Characteristics** | **Flexibility physical activity (d/wk)** | | | | |
| --- | --- | --- | --- | --- | --- |
|  | **0** | **1** | **2** | **3-4** | **≥5**  **(n=5,620)** |
|  | **(n=16,162)** | **(n=3,223)** | **(n=3,817)** | **(n=5,557)** |  |
|  | **N (Percentage^b^)** | | | | |
| **Male** | 6,608 (40.9) | 1,459 (45.3) | 1,499 (39.3) | 2,324 (41.8) | 2,814 (50.1) |
| **Age, years** |  |  |  |  |  |
| 19-49 | 8,202 (50.8) | 2,222 (68.9) | 2,498 (65.4) | 3,296 (59.3) | 2,696 (48.0) |
| 50-59 | 2,803 (17.3) | 571 (17.7) | 751 (19.7) | 1,207 (21.7) | 1,202 (21.4) |
| 60-69 | 2,810 (17.4) | 291 (9.0) | 385 (10.1) | 754 (13.6) | 1,083 (19.3) |
| ≥70 | 2,347 (14.5) | 139 (4.3) | 183 (4.8) | 300 (5.4) | 639 (11.4) |
| **Region^c^** |  |  |  |  |  |
| Metropolitan | 6,661 (41.2) | 1,545 (47.9) | 1,915 (50.2) | 2,743 (49.4) | 2,698 (48.0) |
| Urban | 5,350 (33.1) | 1,168 (36.2) | 1,361 (35.7) | 2,038 (36.7) | 2,004 (35.7) |
| Rural | 4,151 (25.7) | 510 (15.8) | 541 (14.2) | 776 (14.0) | 918 (16.3) |
| **Education attainment** |  |  |  |  |  |
| Lower than high school | 7,059 (43.7) | 670 (20.8) | 833 (21.8) | 1,323 (23.8) | 1,832 (32.6) |
| High school | 5,030 (31.1) | 1,228 (38.1) | 1,528 (40.0) | 2,211 (39.8) | 2,148 (38.2) |
| College or higher | 4,073 (25.2) | 1,325 (41.1) | 1,456 (38.2) | 2,023 (36.4) | 1,640 (29.2) |
| **Occupation** |  |  |  |  |  |
| Nonphysical labor | 2,824 (17.5) | 957 (29.7) | 1,040 (27.3) | 1,397 (25.1) | 1,180 (21.0) |
| Physical labor | 7,142 (44.2) | 1,142 (35.4) | 1,352 (35.4) | 1,974 (35.5) | 2,211 (39.3) |
| Unemployed/homemakers/  students/others | 6,196 (38.3) | 1,124 (34.9) | 1,425 (37.3) | 2,186 (39.3) | 2,229 (39.7) |
| **Household income level** |  |  |  |  |  |
| Quartile 1 | 3,680 (22.8) | 351 (10.9) | 412 (10.8) | 606 (10.9) | 939 (16.7) |
| Quartile 2 | 4,262 (26.4) | 825 (25.6) | 927 (24.3) | 1,305 (23.5) | 1,400 (24.9) |
| Quartile 3 | 4,198 (26.0) | 960 (29.8) | 1,163 (30.5) | 1,627 (29.3) | 1,488 (26.5) |
| Quartile 4 | 3,753 (23.2) | 1,056 (32.8) | 1,262 (33.1) | 1,952 (35.1) | 1,706 (30.4) |
| Missing | 269 (1.7) | 31 (1.0) | 53 (1.4) | 67 (1.2) | 87 (1.6) |
| **Marital status** |  |  |  |  |  |
| Married | 11,709 (72.5) | 2,359 (73.2) | 2,824 (74.0) | 4,286 (77.1) | 4,135 (73.6) |
| Never married | 2,195 (13.6) | 622 (19.3) | 668 (17.5) | 823 (14.8) | 784 (14.0) |
| Divorced/separated/widowed | 2,258 (14.0) | 242 (7.5) | 325 (8.5) | 448 (8.1) | 701 (12.5) |
| **Smoking status** |  |  |  |  |  |
| Never | 10,014 (62.0) | 1,967 (61.0) | 2,492 (65.3) | 3,561 (64.1) | 3,153 (56.1) |
| Former | 2,420 (15.0) | 486 (15.1) | 569 (14.9) | 982 (17.7) | 1,232 (21.9) |
| Current | 3,728 (23.1) | 770 (23.9) | 756 (19.8) | 1,014 (18.3) | 1,235 (22.0) |
| **Alcohol drinking** |  |  |  |  |  |
| Nondrinkers | 4,928 (30.5) | 666 (20.7) | 854 (22.4) | 1,236 (22.2) | 1,442 (25.7) |
| 1 time/month | 4,491 (27.8) | 997 (30.9) | 1,169 (30.6) | 1,750 (31.5) | 1,637 (29.1) |
| ≥2 times/month | 6,743 (41.7) | 1,560 (48.4) | 1,794 (47.0) | 2,571 (46.3) | 2,541 (45.2) |
| **Body Mass Index, kg/m^2^** |  |  |  |  |  |
| <18.5 | 818 (5.1) | 171 (5.3) | 181 (4.7) | 172 (3.1) | 175 (3.1) |
| 18.5-24.9 | 10,181 (63.0) | 2,072 (64.3) | 2,476 (64.9) | 3,670 (66.0) | 3,550 (63.2) |
| ≥25 | 5,163 (32.0) | 980 (30.4) | 1,160 (30.4) | 1,715 (30.9) | 1,895 (33.7) |
| **Self-rated health** |  |  |  |  |  |
| Good | 5,278 (32.7) | 1,204 (37.4) | 1,491 (39.1) | 2,270 (40.9) | 2,531 (45.0) |
| Fair | 7,162 (44.3) | 1,521 (47.2) | 1,767 (46.3) | 2,453 (44.1) | 2,203 (39.2) |
| Poor | 3,722 (23.0) | 498 (15.5) | 559 (14.7) | 834 (15.0) | 886 (15.8) |
| **Walking, h/wk** |  |  |  |  |  |
| 0 | 3,509 (21.7) | 363 (11.3) | 310 (8.1) | 388 (7.0) | 506 (9.0) |
| 0.1-2.4 | 5,002 (31.0) | 1,317 (40.9) | 1,386 (36.3) | 1,640 (29.5) | 1,156 (20.6) |
| 2.5-4.9 | 3,037 (18.8) | 651 (20.2) | 934 (24.5) | 1,580 (28.4) | 1,338 (23.8) |
| 5.0-9.9 | 2,261 (14.0) | 475 (14.7) | 705 (18.4) | 1,118 (20.1) | 1,351 (24.0) |
| ≥10.0 | 2,353 (14.6) | 417 (12.9) | 482 (12.6) | 831 (15.0) | 1,269 (22.6) |
| **Total aerobic physical activity (IPAQ guideline)** | | |  |  |  |
| Low | 7,945 (49.2) | 1,550 (48.1) | 1,486 (38.9) | 1,593 (28.7) | 1,190 (21.2) |
| Moderate | 5,001 (30.9) | 1,043 (32.4) | 1,440 (37.7) | 2,031 (36.6) | 2,107 (37.5) |
| High | 3,216 (19.9) | 630 (19.6) | 891 (23.3) | 1,933 (34.8) | 2,323 (41.3) |
| **Moderate- to vigorous-intensity aerobic physical activity, MET-h/wk** | | | |  |  |
| 0 | 9,916 (61.4) | 1,360 (42.2) | 1,520 (39.8) | 1,960 (35.3) | 2,170 (38.6) |
| 0.1-4.9 | 837 (5.2) | 343 (10.6) | 267 (7.0) | 299 (5.4) | 242 (4.3) |
| 5.0-9.9 | 764 (4.7) | 316 (9.8) | 368 (9.6) | 398 (7.2) | 288 (5.1) |
| 10.0-19.9 | 1,040 (6.4) | 387 (12.0) | 487 (12.8) | 712 (12.8) | 495 (8.8) |
| 20.0-49.9 | 1,647 (10.2) | 484 (15.0) | 736 (19.3) | 1,318 (23.7) | 1,124 (20.0) |
| ≥50.0 | 1,958 (12.1) | 333 (10.3) | 439 (11.5) | 870 (15.7) | 1,301 (23.2) |
| **Muscle-strengthening physical activity, d/wk** | | |  |  |  |
| 0 | 15,212 (94.1) | 2,167 (67.2) | 2,317 (60.7) | 2,787 (50.2) | 2,838 (50.5) |
| 1 | 257 (1.6) | 770 (23.9) | 431 (11.3) | 406 (7.3) | 231 (4.1) |
| 2 | 219 (1.4) | 173 (5.4) | 802 (21.0) | 586 (10.6) | 324 (5.8) |
| 3-4 | 256 (1.6) | 83 (2.6) | 219 (5.7) | 1,618 (29.1) | 600 (10.7) |
| ≥5 | 218 (1.4) | 30 (0.9) | 48 (1.3) | 160 (2.9) | 1,627 (29.0) |

Abbreviation: IPAQ, International Physical Activity Questionnaire; MET, metabolic equivalent task

^a^ When there were less than 1% of missing data, values were imputed with the most frequent categories: education attainment (0.15%, n=50), marital status (0.29%, n=101), smoking status (0.04%, n=13), alcohol drinking (0.24%, n=82), body mass index (0.27% n=92), and self-reported health (0.12%, n=42). Those with missing information on occupation status (0.53%, n=181) and those who reported “unemployed” were combined as the “unemployed/homemakers/students/others” category.

^b^ Values may not sum up to 100% due to rounding.

^c^ Metropolitan includes Seoul special city and 6 other metropolitan cities (i.e. Busan, Daegu, Incheon, Gwangju, Daejeon, and Ulsan). Urban and rural are defined by the legal distribution of submunicipal level divisions based on the size of area and population (urban; ‘Dong’, rural; ‘Eup/Myeon’).

**Supplementary Table 3. Associations of total aerobic physical activity (including walking; MET-h/wk) with all-cause and cause-specific mortality**

|  | **All-cause mortality** | | | | **Cancer mortality** | | | | **Cardiovascular mortality** | |
| --- | --- | --- | --- | --- | --- | --- | --- | --- | --- | --- |
|  | **Death/person-year^a^** | | **HR (95% CI)^b^** | | **Death/person-year^a^** | | **HR (95% CI)^b^** | | **Death/person-year^a^** | **HR (95% CI)^b^** |
| **Total aerobic physical activity (including walking; MET-h/wk)** | | | | | | | | |  |  |
| 0 | | 267/31,115 | | 1.00 (ref) | | 75/31,115 | | 1.00 (ref) | 73/31,115 | 1.00 (ref) |
| 0.1-4.9 | | 172/35,260 | | 0.88 (0.73-1.07) | | 54/35,260 | | 0.95 (0.67-1.35) | 41/35,260 | 0.84 (0.57-1.24) |
| 5.0-9.9 | | 171/36,365 | | 0.85 (0.70-1.04) | | 59/36,365 | | 1.02 (0.72-1.44) | 38/36,365 | 0.75 (0.50-1.11) |
| 10.0-19.9 | | 246/51,935 | | 0.81 (0.68-0.96) | | 93/51,935 | | 1.05 (0.77-1.42) | 53/51,935 | 0.69 (0.48-0.98) |
| 20.0-49.9 | | 384/78,692 | | 0.85 (0.72-0.99) | | 145/78,692 | | 1.07 (0.80-1.42) | 76/78,692 | 0.70 (0.50-0.97) |
| ≥50.0 | | 382/81,859 | | 0.81 (0.69-0.95) | | 148/81,859 | | 1.04 (0.78-1.38) | 57/81,859 | 0.51 (0.35-0.73) |
| *P*-trend^c^ | |  | | 0.02 | |  | | 0.58 |  | <0.001 |

Abbreviation: MET, metabolic equivalent task; HR, Hazard Ratio; CIs, Confidence Intervals

^a^ Person-years may not sum to the total person-years of 315,226 due to rounding.

^b^ Adjusted for sex (male, female), region (metropolitan, urban, rural), education (<high school, high school, college or higher), occupation (nonphysical labor, physical labor, unemployed/homemaker/student/others), household income level (quartile 1,2,3,4, missing), marital status (married, never married, divorced/separated/widowed), smoking status (never, former, current), alcohol drinking (nondrinker, 1 time/month, ≥2 times/month), and self-rated health (good, fair, poor)

^c^ *P*-trend was estimated using the Wald test for continuous trend variable.

**Supplementary Table 4. Associations between aerobic physical activity and all-cause mortality risk, stratified by sex, age, region, income, and BMI**

|  | **Total aerobic physical activity (including walking; IPAQ guideline)^a^** | | | | | |
| --- | --- | --- | --- | --- | --- | --- |
|  | **Low** | | **Moderate** | | **High** | |
|  | **Death/**  **person-year^b^** | **HR (95% CI)^c^** | **Death/**  **person-year ^a^** | **HR (95% CI)^c^** | **Death/**  **person-year ^a^** | **HR (95% CI)^c^** |
| **Stratified by sex** |  |  |  |  |  |  |
| Male  (N=14,704) | 371/46,668 | 1.00 (ref) | 336/43,303 | 0.99 (0.85-1.15) | 261/43,807 | 0.93 (0.79-1.09) |
| Female (N=19,675) | 326/76,873 | 1.00 (ref) | 220/63,253 | 0.91 (0.77-1.08) | 108/41,323 | 0.82 (0.65-1.02) |
| *P*-interaction^d^ | 0.85 | | | | | |
| **Stratified by age group** | | | | | | |
| <65  (N=28,144) | 186/100,498 | 1.00 (ref) | 160/87,691 | 1.01 (0.81-1.25) | 163/73,070 | 1.03 (0.83-1.27) |
| ≥65  (N=6,235) | 511/23,043 | 1.00 (ref) | 396/18,865 | 0.93 (0.81-1.06) | 206/12,059 | 0.80 (0.68-0.94) |
| *P*-interaction^d^ | 0.38 | | | | | |
| **Stratified by region** | | | | | | |
| Urban (N=27,483) | 464/97,979 | 1.00 (ref) | 375/88,175 | 0.94 (0.82-1.09) | 218/65,194 | 0.88 (0.75-1.04) |
| Rural  (N=6,896) | 233/25,561 | 1.00 (ref) | 181/18,381 | 0.99 (0.81-1.20) | 151/19,936 | 0.91 (0.73-1.12) |
| *P*-interaction^d^ | 0.79 | | | | | |
| **Stratified by income^e^** | |  |  |  |  |  |
| Quartile 1, 2 (N=14,707) | 508/54,372 | 1.00 (ref) | 394/43,824 | 0.94 (0.83-1.08) | 236/35,046 | 0.83 (0.71-0.97) |
| Quartile 3, 4 (N=19,165) | 163/67,106 | 1.00 (ref) | 147/60,980 | 1.02 (0.81-1.27) | 118/48,837 | 1.00 (0.79-1.28) |
| *P*-interaction^d^ | 0.72 | | | | | |
| **Stratified by BMI** | |  |  |  |  |  |
| <25.0 kg/m^2^ (N=23,466) | 501/84,640 | 1.00 (ref) | 401/73,722 | 0.93 (0.81-1.07) | 283/56,566 | 0.90 (0.77-1.05) |
| ≥25.0 kg/m^2^ (N=10,913) | 196/38,900 | 1.00 (ref) | 155/32,834 | 1.01 (0.82-1.25) | 86/28,564 | 0.81 (0.63-1.06) |
| *P*-interaction^d^ | 0.47 | | | | | |

Abbreviation: BMI, Body Mass Index; IPAQ, International Physical Activity Questionnaire; CIs, Confidence Intervals; HR, Hazard Ratio

^a^ The “high” category of total aerobic activity included participants with ≥3 days of vigorous-intensity activity achieving total aerobic activity of ≥25 MET-h/wk; or ≥7 days of any combination of walking, moderate-intensity, or vigorous-intensity activities achieving total aerobic physical activity of ≥50 MET-h/wk. The "moderate” category included participants who satisfied at least one of the following 3 criteria: (1) ≥3 days of vigorous-intensity activity of at least 20 min/d; (2) ≥5 days of moderate-intensity activity and/or walking of at least 30 min/d; or (3) ≥5 days of any combination of walking, moderate-intensity, or vigorous-intensity activities achieving total aerobic physical activity of ≥10 MET-h/wk. The “low” category included participant who were not meeting criteria for “moderate” and “high” categories.

^b^ Person-years may not sum to the total person-years of 315,226 due to rounding.

^c^ Adjusted for region (metropolitan, urban, rural), education (<high school, high school, college or higher), occupation (nonphysical labor, physical labor, unemployed/homemaker/student/others), household income (quartiles 1,2,3,4, missing), marital status (married, never married, divorced/separated/widowed), smoking status (never, former, current), alcohol drinking (nondrinker, 1 time/month, ≥2 times/month), and self-rated health (good, fair, poor)

^d^ *P*-interaction was estimated using the Wald test for product term between aerobic physical activity and stratification variable.

^e^ Stratification by income level was conducted after excluding participants with missing income (1.47%, n=507).

**Supplementary Table 5. Associations between muscle-strengthening physical activity and all-cause mortality risk, stratified by sex, age, region, income, and BMI**

|  | **Muscle-strengthening physical activity (d/wk)** | | | | | |
| --- | --- | --- | --- | --- | --- | --- |
|  | **0** | | **1-2** | | **≥3** | |
|  | **Death/**  **person-year^a^** | **HR (95% CI)^b^** | **Death/**  **person-year^a^** | **HR (95% CI)^b^** | **Death/**  **person-year^a^** | **HR (95% CI)^b^** |
| **Stratified by sex** |  |  |  |  |  |  |
| Male  (N=14,704) | 751/81,868 | 1.00 (ref) | 64/22,874 | 0.68 (0.53-0.88) | 153/29,036 | 0.84 (0.70-1.00) |
| Female (N=19,675) | 603/150,497 | 1.00 (ref) | 23/15,332 | 1.02 (0.67-1.56) | 28/15,619 | 0.78 (0.53-1.15) |
| *P*-interaction^c^ | 0.50 | | | | | |
| **Stratified by age group** | | | | | | |
| <65  (N=28,144) | 377/187,284 | 1.00 (ref) | 43/35,396 | 0.69 (0.50-0.95) | 89/38,579 | 0.99 (0.78-1.26) |
| ≥65  (N=6,235) | 977/45,082 | 1.00 (ref) | 44/2,810 | 0.84 (0.62-1.15) | 92/6,075 | 0.69 (0.55-0.87) |
| *P*-interaction^c^ | 0.78 | | | | | |
| **Stratified by region** | | | | | | |
| Urban (N=27,483) | 839/180,381 | 1.00 (ref) | 70/32,630 | 0.78 (0.61-1.00) | 148/38,337 | 0.85 (0.71-1.02) |
| Rural  (N=6,896) | 515/51,984 | 1.00 (ref) | 17/5,576 | 0.66 (0.40-1.07) | 33/6,317 | 0.76 (0.53-1.08) |
| *P*-interaction^c^ | 0.88 | | | | | |
| **Stratified by income^d^** | |  |  |  |  |  |
| Quartile 1, 2 (N=14,707) | 984/103,744 | 1.00 (ref) | 50/13,041 | 0.73 (0.55-0.97) | 104/16,456 | 0.75 (0.61-0.92) |
| Quartile 3, 4 (N=19,165) | 319/124,825 | 1.00 (ref) | 37/24,624 | 0.89 (0.63-1.26) | 72/27,473 | 1.01 (0.77-1.33) |
| *P*-interaction^c^ | 0.69 | | | | | |
| **Stratified by BMI** | |  |  |  |  |  |
| <25.0 kg/m^2^ (N=23,466) | 984/158,990 | 1.00 (ref) | 66/26,181 | 0.78 (0.61-1.01) | 135/29,756 | 0.83 (0.69-1.01) |
| ≥25.0 kg/m^2^ (N=10,913) | 370/73,375 | 1.00 (ref) | 21/12,025 | 0.66 (0.42-1.04) | 46/14,898 | 0.76 (0.55-1.04) |
| *P*-interaction^c^ | 0.36 | | | | | |

Abbreviation: BMI, Body Mass Index; CIs, Confidence Intervals; HR, Hazard Ratio; MET, metabolic equivalent task

^a^ Person-years may not sum to the total person-years of 315,226 due to rounding.

^b^ Adjusted for region (metropolitan, urban, rural), education (<high school, high school, college or higher), occupation (nonphysical labor, physical labor, unemployed/homemaker/student/others), household income level (quartiles 1,2,3,4, missing), marital status (married, never married, divorced/separated/widowed), smoking status (never, former, current), alcohol drinking (nondrinker, 1 time/month, ≥2 times/month), and self-rated health (good, fair, poor)

^c^ *P*-interaction was estimated using the Wald test for product term between muscle-strengthening physical activity and stratification variable.

^d^ Stratification by income level was conducted after excluding participants with missing income (1.47%, n=507).

**Supplementary Table 6. Associations between flexibility physical activity and all-cause mortality risk, stratified by sex, age, region, income, and BMI**

|  | **Flexibility physical activity (d/wk)** | | | | | |
| --- | --- | --- | --- | --- | --- | --- |
|  | **0** | | **1-2** | | **≥3** | |
|  | **Death/**  **person-year^a^** | **HR (95% CI)^b^** | **Death/**  **person-year^a^** | **HR (95% CI)^b^** | **Death/**  **person-year^a^** | **HR (95% CI)^b^** |
| **Stratified by sex** |  |  |  |  |  |  |
| Male  (N=14,704) | 605/60,248 | 1.00 (ref) | 80/26,809 | 0.67 (0.53-0.85) | 283/46,721 | 0.89 (0.77-1.04) |
| Female (N=19,675) | 464/88,488 | 1.00 (ref) | 63/37,019 | 0.76 (0.58-0.99) | 127/55,941 | 0.71 (0.58-0.87) |
| *P*-interaction^c^ | 0.35 | | | | | |
| **Stratified by age group** | | | | | | |
| <65  (N=28,144) | 289/115,094 | 1.00 (ref) | 75/58,606 | 0.78 (0.61-1.02) | 145/87,559 | 0.75 (0.61-0.92) |
| ≥65  (N=6,235) | 780/33,643 | 1.00 (ref) | 68/5,221 | 0.65 (0.51-0.83) | 265/15,103 | 0.86 (0.74-0.99) |
| *P*-interaction^c^ | 0.21 | | | | | |
| **Stratified by region** | | | | | | |
| Urban (N=27,483) | 623/110,244 | 1.00 (ref) | 118/54,156 | 0.78 (0.64-0.95) | 316/86,949 | 0.81 (0.70-0.93) |
| Rural  (N=6,896) | 446/38,493 | 1.00 (ref) | 25/9,672 | 0.51 (0.34-0.77) | 94/15,713 | 0.86 (0.69-1.08) |
| *P*-interaction^c^ | 0.86 | | | | | |
| **Stratified by income^d^** | |  |  |  |  |  |
| Quartile 1, 2 (N=14,707) | 792/72,253 | 1.00 (ref) | 82/22,474 | 0.68 (0.54-0.85) | 264/38,514 | 0.82 (0.71-0.95) |
| Quartile 3, 4 (N=19,165) | 236/73,791 | 1.00 (ref) | 56/40,531 | 0.81 (0.60-1.09) | 136/62,601 | 0.84 (0.68-1.05) |
| *P*-interaction^c^ | 0.50 | | | | | |
| **Stratified by BMI** | |  |  |  |  |  |
| <25.0 kg/m^2^ (N=23,466) | 794/100,811 | 1.00 (ref) | 99/44,508 | 0.67 (0.54-0.82) | 292/69,509 | 0.79 (0.69-0.91) |
| ≥25.0 kg/m^2^ (N=10,913) | 275/47,826 | 1.00 (ref) | 44/19,320 | 0.81 (0.59-1.12) | 118/33,153 | 0.87 (0.69-1.09) |
| *P*-interaction^c^ | 0.35 | | | | | |

Abbreviation: BMI, Body Mass Index; CIs, Confidence Intervals; HR, Hazard Ratio; MET, metabolic equivalent task

^a^ Person-years may not sum to the total person-years of 315,226 due to rounding.

^b^ Adjusted for region (metropolitan, urban, rural), education (<high school, high school, college or higher), occupation (nonphysical labor, physical labor, unemployed/homemaker/student/others), household income level (quartiles 1,2,3,4, missing), marital status (married, never married, divorced/separated/widowed), smoking status (never, former, current), alcohol drinking (nondrinker, 1 time/month, ≥2 times/month), and self-rated health (good, fair, poor)

^c^ *P*-interaction was estimated using the Wald test for product term between flexibility physical activity and stratification variable.

^d^ Stratification by income level was conducted after excluding participants with missing income. (1.47%, n=507)

**Supplementary Table 7. Associations between physical activity types (aerobic, muscle-strengthening, flexibility, meeting guidelines) and all-cause mortality in the 2-y lag analysis**

|  | **Death/person-year^a^** | **Model 1^b^**  **HR (95% CI)** | **Model 2^c^**  **HR (95% CI)** | **Model 3^d^**  **HR (95% CI)** |
| --- | --- | --- | --- | --- |
| **Total aerobic physical activity (including walking; IPAQ guideline)^e^** | | | | |
| Low | 645/96,035 | 1.00 (ref) | 1.00 (ref) | 1.00 (ref) |
| Moderate | 512/83,3327 | 0.95 (0.84-1.07) | 0.95 (0.84-1.06) | 0.94 (0.84-1.06) |
| High | 349/67,152 | 0.90 (0.79-1.03) | 0.90 (0.79-1.02) | 0.90 (0.79-1.03) |
| *P*-trend^e^ |  | 0.12 | 0.10 | 0.10 |
| **Moderate- to vigorous-intensity aerobic physical activity (MET-h/wk)** | | | | |
| 0 | 986/118,813 | 1.00 (ref) | 1.00 (ref) | 1.00 (ref) |
| 0.1-4.9 | 53/14,381 | 0.72 (0.55-0.95) | 0.72 (0.55-0.95) | 0.71 (0.54-0.94) |
| 5.0-9.9 | 49/15,489 | 0.64 (0.48-0.85) | 0.65 (0.48-0.86) | 0.65 (0.49-0.86) |
| 10.0-19.9 | 74/22,417 | 0.68 (0.53-0.86) | 0.68 (0.53-0.86) | 0.68 (0.53-0.86) |
| 20.0-49.9 | 142/38,577 | 0.74 (0.62-0.89) | 0.75 (0.63-0.90) | 0.75 (0.62-0.89) |
| ≥50.0 | 202/36,837 | 0.81 (0.69-0.95) | 0.82 (0.70-0.95) | 0.81 (0.69-0.94) |
| *P*-trend^f^ |  | <0.001 | <0.001 | <0.001 |
| **Muscle-strengthening physical activity (d/wk)** | | | | |
| 0 | 1,259/181,760 | 1.00 (ref) | 1.00 (ref) | 1.00 (ref) |
| 1 | 34/14,908 | 0.66 (0.47-0.94) | 0.67 (0.47-0.95) | 0.66 (0.47-0.94) |
| 2 | 46/14,902 | 0.80 (0.60-1.08) | 0.80 (0.59-1.07) | 0.80 (0.60-1.07) |
| 3-4 | 68/19,769 | 0.78 (0.61-1.01) | 0.78 (0.60-1.01) | 0.77 (0.60-1.00) |
| ≥5 | 99/15,174 | 0.84 (0.68-1.03) | 0.84 (0.68-1.04) | 0.83 (0.67-1.02) |
| *P*-trend^f^ |  | 0.01 | 0.01 | 0.01 |
| **Flexibility physical activity (d/wk)** | | | | |
| 0 | 992/116,442 | 1.00 (ref) | 1.00 (ref) | 1.00 (ref) |
| 1 | 58/22,707 | 0.70 (0.53-0.92) | 0.69 (0.52-0.91) | 0.69 (0.53-0.91) |
| 2 | 75/27,044 | 0.72 (0.57-0.90) | 0.71 (0.56-0.90) | 0.72 (0.57-0.91) |
| 3-4 | 139/39,422 | 0.84 (0.70-1.01) | 0.84 (0.70-1.01) | 0.85 (0.71-1.02) |
| ≥5 | 242/40,897 | 0.80 (0.69-0.93) | 0.80 (0.69-0.93) | 0.80 (0.69-0.93) |
| *P*-trend^f^ |  | <0.001 | <0.001 | <0.001 |
| **Aerobic / muscle-strengthening physical activity guidelines^g^** | | | | |
| Low / <2 d/wk | 591/85,879 | 1.25 (0.99-1.58) |  |  |
| Low / ≥2 d/wk | 54/10,156 | 1.04 (0.74-1.48) |  |  |
| Moderate / <2 d/wk | 436/66,168 | 1.20 (0.95-1.53) |  |  |
| Moderate / ≥2 d/wk | 76/17,159 | 0.98 (0.72-1.33) |  |  |
| High / <2 d/wk | 266/44,622 | 1.14 (0.89-1.47) |  |  |
| High / ≥2 d/wk | 83/22,531 | 1.00 (ref) |  |  |
| *P*-interaction^h^ |  | 0.91 |  |  |

Abbreviation: CIs, Confidence Intervals; HR, Hazard Ratio; IPAQ, International Physical Activity Questionnaire; MET, metabolic equivalent task

^a^ Person-years may not sum to the total person-years of 246,514 due to rounding.

^b^ Model 1 includes sex (male, female), region (metropolitan, urban, rural), education (<high school, high school,

college or higher), occupation (nonphysical labor, physical labor, unemployed/homemaker/student/others), household income level (quartiles 1,2,3,4, missing), marital status (married, never married, divorced/separated/widowed), smoking status (never, former, current), alcohol drinking (nondrinker, 1 time/month, ≥2 times/month), and self-rated health (good, fair, poor).

^c^ Model 2 includes all variables in Model 1 plus body mass index (<18.5, 18.5-24.9, ≥25.0 kg/m^2^).

^d^ Model 3 includes all variables in Model 2 plus prevalence of cardiovascular disease risk factors (type 2 diabetes, hypertension, dyslipidemia).

^e^ The “high” category of total aerobic activity included participants with ≥3 days of vigorous-intensity activity achieving total aerobic activity of ≥25 MET-h/wk; or ≥7 days of any combination of walking, moderate-intensity, or vigorous-intensity activities achieving total aerobic physical activity of ≥50 MET-h/wk. The "moderate” category included participants who satisfied at least one of the following 3 criteria: (1) ≥3 days of vigorous-intensity activity of at least 20 min/d; (2) ≥5 days of moderate-intensity activity and/or walking of at least 30 min/d; or (3) ≥5 days of any combination of walking, moderate-intensity, or vigorous-intensity activities achieving total aerobic physical activity of ≥10 MET-h/wk. The “low” category included participant who were not meeting criteria for “moderate” and “high” categories.

^f^ *P*-trend test was estimated using the Wald test for continuous trend variable.

^g^ 2-y lag analysis for physical activity guidelines was conducted in the model 1 only.

^h^ *P*-interaction was estimated using the Wald test for product term between aerobic physical activity (low, moderate, high) and muscle-strengthening physical activity (<2, ≥2).

**Supplementary Table 8. Associations between three physical activity types (aerobic, muscle-strengthening, flexibility, meeting guidelines) and cause-specific mortality (cancer- and cardiovascular disease-specific mortality) in the 2-y lag analysis**

|  | **Cancer mortality** | | **Cardiovascular mortality** | |
| --- | --- | --- | --- | --- |
|  | **Death/person-year^a^** | **HR (95% CI)^b^** | **Death/person-year^a^** | **HR (95% CI)^b^** |
| **Total aerobic physical activity (including walking; IPAQ guideline)** | | | | |
| Low | 214/96,035 | 1.00 (ref) | 155/96,035 | 1.00 (ref) |
| Moderate | 189/83,327 | 1.04 (0.85-1.27) | 110/83,327 | 0.87 (0.68-1.12) |
| High | 132/67,152 | 0.97 (0.77-1.21) | 47/67,152 | 0.54 (0.39-0.76) |
| *P*-trend^c^ |  | 0.83 |  | 0.77 |
| **Moderate- to vigorous-intensity aerobic physical activity (MET-h/wk)** | | | | |
| 0 | 332/118,813 | 1.00 (ref) | 231/118,813 | 1.00 (ref) |
| 0.1-4.9 | 18/14,381 | 0.73 (0.45-1.17) | -^d^/14,381 | 0.56 (0.29-1.10) |
| 5.0-9.9 | 20/15,489 | 0.76 (0.49-1.20) | -^d^ /15,489 | 0.48 (0.24-0.97) |
| 10.0-19.9 | 27/22,417 | 0.69 (0.47-1.03) | 14/22,417 | 0.59 (0.34-1.02) |
| 20.0-49.9 | 54/38,577 | 0.78 (0.58-1.04) | 21/38,577 | 0.54 (0.34-0.83) |
| ≥50.0 | 84/36,837 | 0.95 (0.74-1.22) | 29/36,837 | 0.53 (0.35-0.78) |
| *P*-trend^c^ |  | 0.17 |  | <0.001 |
| **Muscle-strengthening physical activity (d/wk)** | | | | |
| 0 | 424/181,760 | 1.00 (ref) | 270/181,760 | 1.00 (ref) |
| 1-2 | 42/29,810 | 1.04 (0.75-1.46) | 11/29,810 | 0.62 (0.34-1.14) |
| 3-4 | 32/19,769 | 0.99 (0.68-1.43) | 11/19,769 | 0.79 (0.43-1.47) |
| ≥5 | 37/15,174 | 0.86 (0.61-1.22) | 20/15,174 | 0.97 (0.61-1.55) |
| *P*-trend^c^ |  | 0.49 |  | 0.55 |
| **Flexibility physical activity (d/wk)** | | | | |
| 0 | 331/116,442 | 1.00 (ref) | 225/116,442 | 1.00 (ref) |
| 1-2 | 57/49,752 | 0.85 (0.64-1.13) | 17/49,752 | 0.46 (0.28-0.76) |
| 3-4 | 59/39,422 | 0.94 (0.70-1.25) | 21/39,422 | 0.67 (0.42-1.05) |
| ≥5 | 88/40,897 | 0.81 (0.64-1.03) | 49/40,897 | 0.79 (0.57-1.10) |
| *P*-trend^c^ |  | 0.10 |  | 0.07 |
| **Aerobic / muscle-strengthening physical activity guidelines** | | | | |
| Low / <2 d/wk | 193/85,879 | 1.06 (0.73-1.53) | 142/85,879 | 1.87 (0.99-3.56) |
| Low / ≥2 d/wk | 21/10,156 | 0.97 (0.56-1.67) | 13/10,156 | 1.93 (0.85-4.40) |
| Moderate / <2 d/wk | 155/66,168 | 1.11 (0.76-1.62) | 94/66,168 | 1.64 (0.85-3.15) |
| Moderate / ≥2 d/wk | 34/17,159 | 1.00 (0.62-1.60) | 16/17,159 | 1.64 (0.76-3.54) |
| High / <2 d/wk | 96/44,622 | 1.02 (0.69-1.50) | 37/44,622 | 1.02 (0.51-2.06) |
| High / ≥2 d/wk | 36/22,531 | 1.00 (ref) | 10/22,531 | 1.00 (ref) |
| *P*-interaction^e^ |  | <0.001 |  | 0.01 |

Abbreviation: CIs, Confidence Intervals; HR, Hazard Ratio; IPAQ, International Physical Activity Questionnaire; MET, metabolic equivalent task

^a^ Person-years may not sum to the total person-years of 246,514 due to rounding.

^b^ Adjusted for sex (male, female), region (metropolitan, urban, rural), education (<high school, high school, college or higher), occupation (nonphysical labor, physical labor, unemployed/homemaker/student/others), household income level (quartiles 1,2,3,4, missing), marital status (married, never married, divorced/separated/widowed), smoking status (never, former, current), alcohol drinking (nondrinker, 1 time/month, ≥2 times/month), and self-rated health (good, fair, poor).

^c^ *P*-trend was estimated using the Wald test for continuous trend variable.

^d^ Estimates for death count <10 is not disclosed.

^e^ *P*-interaction was estimated using the Wald test for product term between aerobic physical activity (low, moderate, high) and muscle-strengthening physical activity (<2, ≥2).

**Supplementary Table 9. Associations between three physical activity types (aerobic, muscle-strengthening, flexibility, meeting guidelines) and all-cause mortality in the 5-y lag analysis**

|  | **Death/person-year^a^** | | **Model 1^b^**  **HR (95% CI)** | | **Model 2^c^**  **HR (95% CI)** | | **Model 3^d^**  **HR (95% CI)** | |
| --- | --- | --- | --- | --- | --- | --- | --- | --- |
| **Total aerobic physical activity (including walking; IPAQ guideline)** | | | | | | | | |
| Low | | 414/55,207 | | 1.00 (ref) | | 1.00 (ref) | | 1.00 (ref) |
| Moderate | | 345/48,835 | | 0.95 (0.83-1.10) | | 0.95 (0.82-1.10) | | 0.95 (0.82-1.10) |
| High | | 246/40,376 | | 0.94 (0.80-1.11) | | 0.93 (0.79-1.10) | | 0.93 (0.79-1.10) |
| *P*-trend^e^ | |  | | 0.43 | | 0.37 | | 0.39 |
| **Moderate- to vigorous-intensity aerobic physical activity (MET-h/wk)** | | | | | | | | |
| 0 | | 647/68,717 | | 1.00 (ref) | | 1.00 (ref) | | 1.00 (ref) |
| 0.1-4.9 | | 44/8,458 | | 0.90 (0.66-1.22) | | 0.90 (0.66-1.22) | | 0.89 (0.65-1.21) |
| 5.0-9.9 | | 34/91,21 | | 0.66 (0.47-0.93) | | 0.67 (0.47-0.94) | | 0.66 (0.47-0.93) |
| 10.0-19.9 | | 47/13,114 | | 0.65 (0.49-0.88) | | 0.65 (0.48-0.88) | | 0.65 (0.48-0.88) |
| 20.0-49.9 | | 98/22,741 | | 0.76 (0.61-0.95) | | 0.77 (0.62-0.95) | | 0.76 (0.61-0.94) |
| ≥50.0 | | 135/22,267 | | 0.79 (0.65-0.96) | | 0.79 (0.65-0.96) | | 0.78 (0.65-0.95) |
| *P*-trend^e^ | |  | | <0.001 | | <0.001 | | <0.001 |
| **Muscle-strengthening physical activity (d/wk)** | | | | | | | | |
| 0 | | 847/106,652 | | 1.00 (ref) | | 1.00 (ref) | | 1.00 (ref) |
| 1 | | 16/8,651 | | 0.49 (0.30-0.81) | | 0.50 (0.30-0.82) | | 0.49 (0.30-0.81) |
| 2 | | 26/8,632 | | 0.72 (0.48-1.06) | | 0.71 (0.48-1.05) | | 0.71 (0.48-1.05) |
| 3-4 | | 45/11,493 | | 0.78 (0.57-1.07) | | 0.78 (0.57-1.07) | | 0.78 (0.57-1.06) |
| ≥5 | | 71/8,990 | | 0.88 (0.69-1.13) | | 0.89 (0.69-1.14) | | 0.87 (0.68-1.11) |
| *P*-trend^e^ | |  | | 0.06 | | 0.06 | | 0.05 |
| **Flexibility physical activity (d/wk)** | | | | | | | | |
| 0 | | 664/68,649 | | 1.00 (ref) | | 1.00 (ref) | | 1.00 (ref) |
| 1 | | 30/13,082 | | 0.57 (0.39-0.82) | | 0.56 (0.39-0.82) | | 0.56 (0.39-0.82) |
| 2 | | 53/15,639 | | 0.79 (0.60-1.04) | | 0.78 (0.59-1.03) | | 0.79 (0.60-1.04) |
| 3-4 | | 92/22,845 | | 0.87 (0.69-1.08) | | 0.86 (0.69-1.08) | | 0.87 (0.70-1.09) |
| ≥5 | | 166/24,203 | | 0.80 (0.67-0.95) | | 0.80 (0.67-0.96) | | 0.80 (0.67-0.95) |
| *P*-trend^e^ | |  | | 0.01 | | 0.01 | | 0.01 |
| **Aerobic / muscle-strengthening physical activity guidelines^f^** | | | | | | | | |
| Low / <2 d/wk | | 383/49,344 | | 1.18 (0.89-1.57) | |  | |  |
| Low / ≥2 d/wk | | 31/5,864 | | 0.88 (0.57-1.37) | |  | |  |
| Moderate / <2 d/wk | | 293/38,875 | | 1.12 (0.84-1.49) | |  | |  |
| Moderate / ≥2 d/wk | | 52/9,960 | | 0.97 (0.67-1.41) | |  | |  |
| High / <2 d/wk | | 187/27,084 | | 1.10 (0.82-1.49) | |  | |  |
| High / ≥2 d/wk | | 59/13,292 | | 1.00 (ref) | |  | |  |
| *P*-interaction^g^ | |  | | 0.72 | |  | |  |

Abbreviation: CIs, Confidence Intervals; HR, Hazard Ratio; IPAQ, International Physical Activity Questionnaire; MET, metabolic equivalent task

^a^ Person-years may not sum to the total person-years of 144,418 due to rounding.

^b^ Model 1 includes sex (male, female), region (metropolitan, urban, rural), education (<high school, high school,

college or higher), occupation (nonphysical labor, physical labor, unemployed/homemaker/student/others), household income level (quartiles 1,2,3,4, missing), marital status (married, never married, divorced/separated/widowed), smoking status (never, former, current), alcohol drinking (nondrinker, 1 time/month, ≥2 times/month), and self-rated health (good, fair, poor).

^c^ Model 2 includes all variables in Model 1 plus body mass index (<18.5, 18.5-24.9, ≥25.0 kg/m^2^).

^d^ Model 3 includes all variables in Model 2 plus prevalence of cardiovascular disease risk factors (type 2 diabetes, hypertension, dyslipidemia).

^e^ *P*-trend was estimated using the Wald test for continuous trend variable.

^f^ 5-y lag analysis for physical activity guidelines was conducted in the model 1 only.

^g^ *P*-interaction was estimated using the Wald test for product term between aerobic physical activity (low, moderate, high) and muscle-strengthening physical activity (<2, ≥2).

**Supplementary Table 10. Associations between three physical activity types (aerobic, muscle-strengthening, flexibility, meeting guidelines) and cause-specific mortality (cancer- and cardiovascular disease-specific mortality) in the 5-y lag analysis**

|  | **Cancer mortality** | | **Cardiovascular mortality** | |
| --- | --- | --- | --- | --- |
|  | **Death/person-year^a^** | **HR (95% CI)^b^** | **Death/person-year^a^** | **HR (95% CI)^b^** |
| **Total aerobic physical activity (including walking; IPAQ guideline)** | | | | |
| Low | 131/55,207 | 1.00 (ref) | 103/55,207 | 1.00 (ref) |
| Moderate | 129/48,835 | 1.10 (0.86-1.41) | 77/48,835 | 0.89 (0.66-1.19) |
| High | 90/40,376 | 1.02 (0.77-1.34) | 35/40,376 | 0.58 (0.38-0.86) |
| *P*-trend^c^ |  | 0.81 |  | 0.01 |
| **Moderate- to vigorous-intensity aerobic physical activity (MET-h/wk)** | | | | |
| 0 | 209/68,177 | 1.00 (ref) | 154/68,177 | 1.00 (ref) |
| 0.1-4.9 | 15/8,458 | 0.94 (0.56-1.60) | -^d^ /8,458 | 0.73 (0.36-1.50) |
| 5.0-9.9 | 16/9,121 | 0.92 (0.55-1.55) | -^d^ /9,121 | 0.64 (0.30-1.36) |
| 10.0-19.9 | 18/13,114 | 0.72 (0.44-1.17) | 10/13,114 | 0.64 (0.34-1.21) |
| 20.0-49.9 | 37/22,741 | 0.80 (0.56-1.14) | 16/22,741 | 0.61 (0.36-1.01) |
| ≥50.0 | 55/22,267 | 0.95 (0.69-1.29) | 20/22,267 | 0.52 (0.32-0.84) |
| *P*-trend^c^ |  | 0.28 |  | <0.001 |
| **Muscle-strengthening physical activity (d/wk)** | | | | |
| 0 | 283/106,652 | 1.00 (ref) | 179/106,652 | 1.00 (ref) |
| 1-2 | 19/17,282 | 0.71 (0.44-1.16) | -^d^ /17,282 | 0.82 (0.41-1.65) |
| 3-4 | 22/11,493 | 0.97 (0.62-1.52) | -^d^ /11,493 | 0.91 (0.44-1.88) |
| ≥5 | 26/8,990 | 0.86 (0.56-1.30) | 19/8,990 | 1.42 (0.87-2.31) |
| *P*-trend^c^ |  | 0.41 |  | 0.32 |
| **Flexibility physical activity (d/wk)** | | | | |
| 0 | 218/68,649 | 1.00 (ref) | 149/68,649 | 1.00 (ref) |
| 1-2 | 33/28,721 | 0.75 (0.52-1.08) | 14/28,721 | 0.60 (0.34-1.05) |
| 3-4 | 38/22,845 | 0.88 (0.62-1.27) | 16/22,845 | 0.84 (0.50-1.43) |
| ≥5 | 61/24,203 | 0.80 (0.60-1.07) | 36/24,203 | 0.89 (0.61-1.30) |
| *P*-trend^c^ |  | 0.12 |  | 0.44 |
| **Aerobic / muscle-strengthening physical activity guidelines** | | | | |
| Low / <2 d/wk | 119/49,344 | 1.02 (0.65-1.60) | 94/49,344 | 1.58 (0.76-3.27) |
| Low / ≥2 d/wk | 12/5,864 | 0.85 (0.42-1.71) | -^d^ /5,864 | 1.71 (0.65-4.45) |
| Moderate / <2 d/wk | 107/38,875 | 1.13 (0.72-1.79) | 61/38,875 | 1.30 (0.62-2.73) |
| Moderate / ≥2 d/wk | 22/9,960 | 0.95 (0.53-1.70) | 16/9,960 | 2.13 (0.93-4.87) |
| High / <2 d/wk | 65/27,084 | 1.02 (0.63-1.63) | 27/27,084 | 0.90 (0.41-1.98) |
| High / ≥2 d/wk | 25/13,292 | 1.00 (ref) | -^d^ /13,292 | 1.00 (ref) |
| *P*-interaction^e^ |  | <0.001 |  | 0.04 |

Abbreviation: CIs, Confidence Intervals; HR, Hazard Ratio; IPAQ, International Physical Activity Questionnaire; MET, metabolic equivalent task^a^ Person-years may not sum to the total person-years of 144,418 due to rounding.

^b^ Adjusted for sex (male, female), region (metropolitan, urban, rural), education (<high school, high school, college or higher), occupation (nonphysical labor, physical labor, unemployed/homemaker/student/others), household income level (quartiles 1,2,3,4, missing), marital status (married, never married, divorced/separated/widowed), smoking status (never, former, current), alcohol drinking (nondrinker, 1 time/month, ≥2 times/month), and self-rated health (good, fair, poor).

^c^ *P*-trend was estimated using the Wald test for continuous trend variable.

^d^ Estimates for death count <10 is not disclosed.

^e^ *P*-interaction was estimated using the Wald test for product term between aerobic physical activity (low, moderate, high) and muscle-strengthening physical activity (<2, ≥2).
